# Liposomes or Extracellular Vesicles: A Comprehensive Comparison of Both Lipid Bilayer Vesicles for Pulmonary Drug Delivery

**DOI:** 10.3390/polym15020318

**Published:** 2023-01-07

**Authors:** Ali Al-Jipouri, Samah Hamed Almurisi, Khater Al-Japairai, Latifah Munirah Bakar, Abd Almonem Doolaanea

**Affiliations:** 1 Institute for Transfusion Medicine, University Hospital Essen, University of Duisburg-Essen, D-45147 Essen, Germany; 2 Department of Pharmaceutical Technology, Kulliyyah of Pharmacy, International Islamic University Malaysia, Kuantan 25200, Malaysia; 3 Department of Pharmaceutical Engineering, Faculty of Chemical and Process Engineering Technology, Universiti Malaysia Pahang, Gambang 26300, Malaysia; 4 Faculty of Applied Sciences, Universiti Teknologi MARA (UiTM) Selangor, Shah Alam 40450, Malaysia; 5 Department of Pharmaceutical Technology, Faculty of Pharmacy, University College MAIWP International (UCMI), Kuala Lumpur 68100, Malaysia

**Keywords:** liposomes, extracellular vesicles, hybrid vesicles, pulmonary drug delivery, inhalers, nebulizers, scale-up

## Abstract

The rapid and non-invasive pulmonary drug delivery (PDD) has attracted great attention compared to the other routes. However, nanoparticle platforms, like liposomes (LPs) and extracellular vesicles (EVs), require extensive reformulation to suit the requirements of PDD. LPs are artificial vesicles composed of lipid bilayers capable of encapsulating hydrophilic and hydrophobic substances, whereas EVs are natural vesicles secreted by cells. Additionally, novel LPs-EVs hybrid vesicles may confer the best of both. The preparation methods of EVs are distinguished from LPs since they rely mainly on extraction and purification, whereas the LPs are synthesized from their basic ingredients. Similarly, drug loading methods into/onto EVs are distinguished whereby they are cell- or non-cell-based, whereas LPs are loaded via passive or active approaches. This review discusses the progress in LPs and EVs as well as hybrid vesicles with a special focus on PDD. It also provides a perspective comparison between LPs and EVs from various aspects (composition, preparation/extraction, drug loading, and large-scale manufacturing) as well as the future prospects for inhaled therapeutics. In addition, it discusses the challenges that may be encountered in scaling up the production and presents our view regarding the clinical translation of the laboratory findings into commercial products.

## 1. Introduction

**Figure 1:**
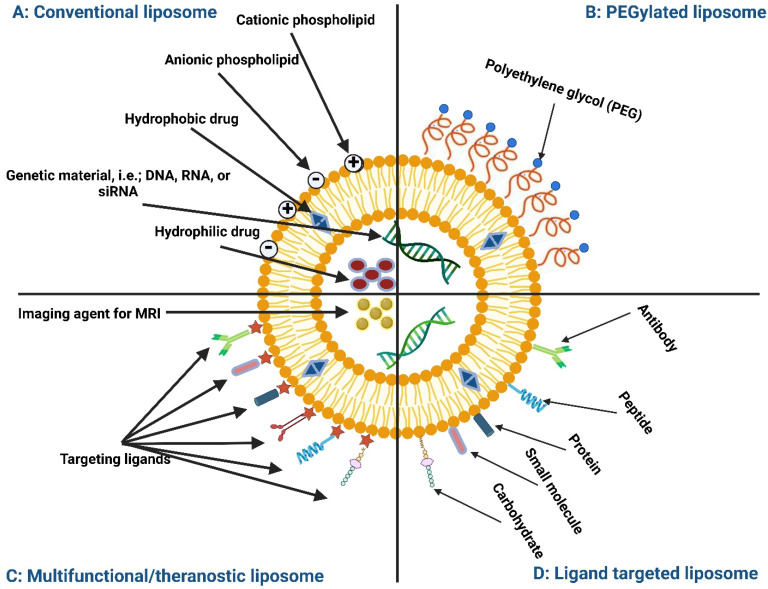
Liposome classification according to the surface modification strategy: (**A**) Conventional liposomes. (**B**) PEGylated/stealth liposomes. (**C**) Multifunctional/theranostic liposomes. (**D**) Ligand targeted liposomes.

Pulmonary drug delivery (PDD) is primarily used to treat acute respiratory tract infections as well as chronic respiratory disorders (CRDs); the most common are asthma, chronic obstructive pulmonary disease (COPD), tuberculosis (TB), and lung cancer [1]. PDD is also used to treat systemic diseases such as diabetes by delivering insulin into the alveolar region for fast absorption into the general circulation. This is owing to the large surface area of human lungs (70–100 m^2^) and the extremely thin (0.1–0.2 µm) mucosal membrane of the lung epithelium, which enhances drug absorption to the systemic circulation [2]. In PDD, the drugs can be delivered directly to their site of action, thus leading to advantages such as a small amount of drug being needed, less adverse reactions, and rapid onset of action [3]. Paul Ehrlich proposed the concept of the “magic bullet,” a drug that selectively targets the disease cells without affecting normal cells [4]. The concept of the “magic bullet” is made possible through the use of nanoparticles. An ideal nanoparticle system should avoid the premature release of the drug before reaching the site of action and release the drug directly where it exerts its efficacy [5]. Amongst several types of nanoparticle delivery systems, lipid bilayer vesicles like liposomes (LPs) are versatile drug packaging and delivery platforms discovered in the 1960s. LPs are composed of an aqueous core, into which hydrophilic drugs can be embedded, surrounded by a lipid bilayer where hydrophobic drugs can be incorporated. Advances in LPs led to the fabrication of LPs with different surface characteristics to suit different applications, including treatment and diagnosis (Figure 1). Although LPs enhance the protection of encapsulated drugs against degradation in circulation, their clinical applications face significant biological barriers to be overcome. These barriers include the rapid removal from the circulation, off-target accumulation in the filtering organs, and stimulation of the innate immune response [6].

**Figure 2:**
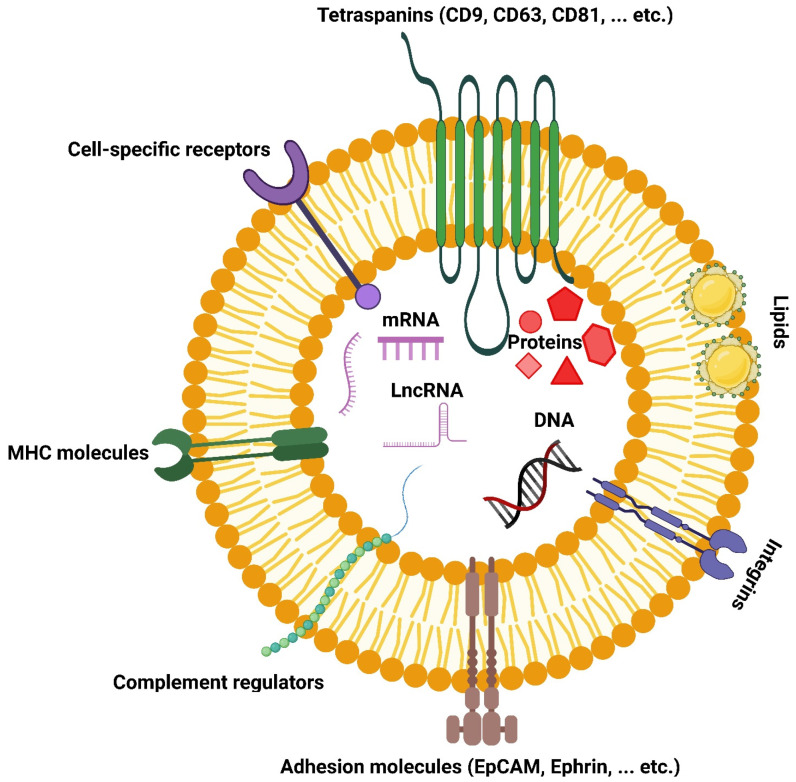
Structure and composition of extracellular vesicles (EVs).

**Figure 3:**
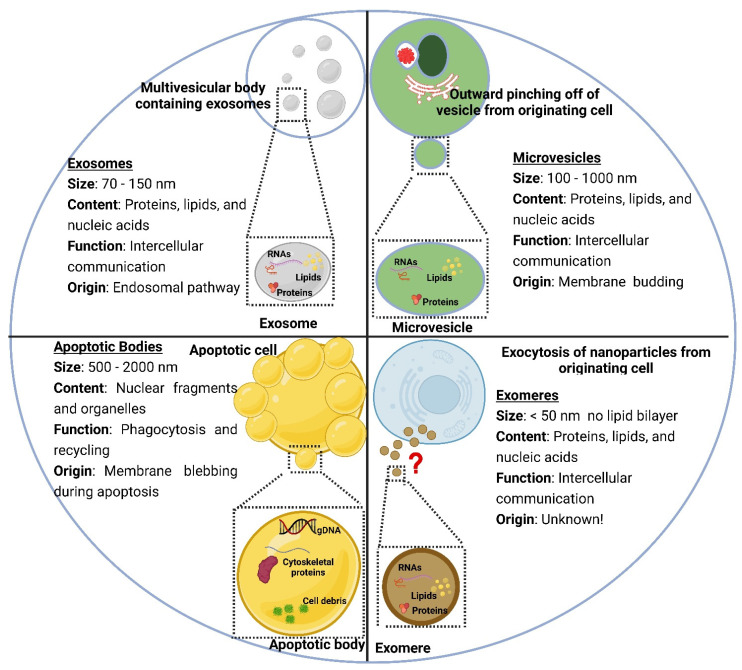
Major subtypes of extracellular vesicles (EVs).

Compared to synthetic LPs, extracellular vesicles (EVs) have emerged as a more complex form of lipid bilayer vesicles extracted from biological fluids. EVs were first described by the American neurochemist Eberhard G. Trams in 1981 [7] (Figure 2). Later in 1983, Johnston and colleagues described EVs’ necessity for the function and homeostasis of multicellular organisms [8]. Since 1996, EVs have been recognized as essential intercellular biomolecule carriers/exchangers that support important cellular functions [9]. The term “EVs” is used to refer to all types of cell-derived membrane vesicles because no consensus has been reached yet regarding the isolation and detection techniques for the accurate separation of these vesicles’ subpopulations [10]. However, based on their intracellular origin, biogenesis, physicochemical properties, and surface markers, EVs are commonly classified into exosomes (70–150 nm), microvesicles (100–1000 nm), and apoptotic bodies (500–2000 nm) [11]. In addition, ‘exomeres,’ a recently discovered subpopulation of EVs, are tiny non-membranous nanovesicles (<50 nm) secreted by cells (Figure 3). The exact mechanism for the biogenesis and secretion of exomeres is unclear. Different from the other subtypes of EVs, exomeres are not enveloped by lipid bilayer membranes and are significantly smaller [12,13]. By harnessing their intrinsic tissue-homing capabilities, EVs have been explored to deliver therapeutic payloads to specific cells or tissues [14]. Exosomes are believed to be the ideal candidates of defined size and function for drug delivery [15]. EVs are structurally comparable to LPs, given that both are phospholipid-based vesicles.

The main components of LPs and EVs are phospholipids (PLs), a class of natural and bioinspired excipients (NBEs) endogenous to the human body and a primary constituent of cell membranes and pulmonary surfactants. However, EVs are more complex due to their biological origin and similarity in composition to their generating cells. Thus, EVs fulfill the complexity requirement for obtaining the optimal biological level of nanomedicine carriers, as they contain up to hundreds of different types of lipids, proteins, and carbohydrates, as well as internal cargo and surface-associated molecules [16,17]. Although EVs are unique protein-decorated phospholipid vesicles, their superiority over engineered LPs for drug delivery and the associated risks against benefits need extensive studies [18]. While LPs and EVs are increasingly considered therapeutics for a variety of diseases [19,20], the development of inhaled therapies, especially biopharmaceuticals, is challenging [21]. Airway architecture, moisture, mucociliary clearance, and alveolar macrophages are essential to maintain lung sterility and thus constitute critical barriers to the therapeutic efficacy of inhaled formulations. In addition, inhaled biopharmaceutical formulations should have favorable biophysical characteristics to overcome the stresses during production, transportation, and aerosolization. Furthermore, to achieve systemic therapy, they need to cross the lung epithelium to reach the general circulation at sufficient concentrations [22]. Solvent, buffer, and pH are all examples of formulation factors that can affect the stability, aggregation, sedimentation, and leakage of the drug from the vesicles during storage [23]. LPs are chemically unstable when stored as aqueous dispersions because PLs are susceptible to peroxidation and hydrolysis, resulting in increased permeability [24]. Physically, LPs stability considerations include appearance, size, and size distribution, which can be examined using transmission electron microscopy (TEM) and dynamic light scattering (DLS) [25]. Freeze-drying, spray-drying, and spray-freeze drying are among several techniques to improve the physicochemical stability of the LPs [26].

When considering PDD, not only is the formulation important, but the type of inhaler is also critical. The commonly used inhalers include pressurized metered dose inhalers (pMDIs), dry powder inhalers (DPIs), soft mist inhalers (SMIs), and nebulizers. Although pMDIs are multi-dose inhalers containing drugs as solutions or suspensions in one propellant or propellant blend, biological drugs generally suffer a stability issue due to the denaturation upon aerosolization and inappropriate particle distribution. DPIs are pre-metered doses in capsules/blisters or self-dose of loose powder, where the drug is inhaled either pure or blended with a carrier via the patient’s generated airflow. Bio-inhalers require biological formulation into successfully stabilized dry inhalable powders. SMIs are novel multidose propellant-free liquid inhalers where aqueous formulations are forced through nozzles similar to nebulizers, and they are preferable for biologics delivery than pMDIs. For pulmonary administration of biologics, nebulizers are the most commonly used devices owing to their suitability for patients of all ages and of different disease stages. However, not all nebulizers (jet, ultrasonic, and vibrating-mesh) are equally suitable for protein delivery since the problem related to the large air-liquid interface (24–1500 m^2^) interferes with protein delivery and causes denaturation in a multi-step process [27]. 

In this review, we critically evaluated the advantages and disadvantages of LPs and EVs in the context of PDD. We identified the advantages of EVs as next-generation therapeutics over standard delivery methods; discussed current obstacles related to their clinical and industrial translation. We also elaborated on the critical composition, such as lipids and proteins, as well as the engineering potential of both LPs and EVs to enhance pharmacokinetic properties suitable for serving as PDD. In addition, we explored the employment of EVs for the pulmonary delivery of small molecules, proteins, and nucleic acids and made a comparison with LPs. The gist of this discussion is to attempt to embrace a vision of how a hybrid vesicle, using only basic constituents, can confer the best of both LPs and EVs in PDD.

## 2. Structure and Composition of Liposomes and Extracellular Vesicles

### 2.1. Structure of Liposomes and Extracellular Vesicles

In terms of size, LPs are categorized as small vesicles between 20–100 nm, large vesicles between 100–1000 nm, and giant vesicles larger than 1000 nm [28]. In terms of lamellarity, i.e., internal lipid structures within a lipid bilayer vesicle, LPs are categorized as unilamellar-consisting of a single outer lipid bilayer, multilamellar-in which consecutive, concentric lipid bilayers are present within a single outer lipid bilayer, and multivesicular-where separate, smaller sized vesicles are contained within a single outer lipid bilayer [29]. In terms of vesicles, LPs are categorized as small unilamellar vesicles (SUVs), large unilamellar vesicles (LUVs), giant unilamellar vesicles (GUVs), and multilamellar vesicles (MLVs). Lamellarity can be a factor that influences the efficiency of cargo encapsulation, release, and fate following intracellular uptake [30].

EVs are secreted by cells as nano-size lipid bilayer vesicles. EVs contain several components from the secreting cell, such as transmembrane proteins, RNA, DNA, and cytosolic content [31]. However, EVs were recently found to function as a complex intercellular communication system that is present in several living organisms such as plants, archaea, fungi, and bacteria [32,33]. The content of EVs varies based on the producing cell and the physiological condition like differentiation and immune response. The protein content varies based on the microenvironment of the secreting cell and the method of isolation used. The most commonly used proteins to characterize EVs are CD9, CD63, and CD81 [34,35]. In addition, EVs contain almost all RNA types ranging from 25 to 700 nucleotides, whereby small RNA molecules from normal and tumor cells are especially abundant [36]. RNA content also varies based on the secreting cell and EVs function. For example, miRNA amount is higher in senescent cells’ EVs and seems to have an anti-apoptotic function [37]. EVs content of larger RNA molecules (mRNA) has several 3-untranslated regions rich in miRNA binding sites that may compete with the receiving cell RNA, thereby regulating the receiving cell transcriptional activity [35,38].

### 2.2. Lipid Composition

PLs are available in a wide range of lipid head groups/charges and chain lengths/saturations from both synthetic and natural sources. Glycerophospholipids, which are amphiphilic lipids composed of a glycerol molecule bound to a phosphate group and two fatty acid chains, are the major component of LPs [39]. These are the example of natural glycerophospholipids which found abundant in nature: phosphatidic acid (PA), phosphatidylcholine (PC), phosphatidylethanolamine (PE), phosphatidylglycerol (PG), phosphatidylinositol (PI), phosphatidylserine (PS) and cardiolipin (CL) [40]. Meanwhile, examples of synthetic lipids are dimyristoyl phosphatidylcholine (DMPC), 1,2-dioleoyl-sn-glycero-3-phosphocholine (DOPC), dipalmitoyl phosphatidylcholine (DPPC), 1,2-dipalmitoyl-sn-glycero-3-phosphoglycerol (DPPG), 1,2-distearoyl-sn-glycero-3-phosphate (DSPA), distearoyl phosphatidylcholine (DSPC), 1,2-distearoyl-sn-glycero-3-phospho-(1′-rac-glycerol) (DSPG), hydrogenated soy phosphatidylcholine (HSPC), 1-palmitoyl-2-oleoyl-sn-glycero-3-phosphocholine (POPC), and (1-palmitoyl-2-stearoyl(5-DOXYL)-sn-glycero-3phosphocholine (SLPC) [41].

LPs made from cationic lipids such as 1,2-dioleoyl-3-dimethylammonium-propane (DODAP) and 1,2-dioleoyl-3-trimethylammonium-propane (DOTAP) could facilitate electrostatic interactions with negatively charged DNA and the cell membrane [41]. Negatively charged LPs are more similar to cell/EVs membranes such as PG, PS, PI, and PA [41]. Neutral LPs are made from zwitterionic lipids [25]. Similar to LPs, EVs exist in various forms, such as single and double vesicles as well as double- and multi-layered vesicles [42]. Extensive lipidomic analyses showed that EVs contain lipid species also present in the cell’s plasma membrane, rich in glycosphingolipids, sphingomyelins, PE, PS, PC, and cholesterol [43]. The selection of lipids allows the generation of numerous LPs compositions, while cholesterol addition rigidifies the lipid bilayer and stabilizes the structure. 

The complexity of EVs lipid profiles comes from the type of the lipid head group/charge and fatty acid tail length/saturation [44]. Similar to LPs, the lipid bilayer of EVs has a highly curved structure rich in lipid molecules that allows (1) positive curvature in the outer membrane like one fatty acid chain lipids, and (2) negative curvature in the inner membrane like more protruding fatty acid chains lipids [45]. Standard procedures used to manufacture LPs generate a random mixture of lipids in their lipid bilayers, where the asymmetric distribution of lipid species is not possible. As with LPs, the lipids of EVs can be categorized as anionic (e.g., glycerophosphatidic acid, glycerophosphoglycerol, glycerophosphoinositol), weakly anionic (e.g., ceramide and glycerophosphoethanolamine) or neutral (e.g., mono-, di-, and triacylglycerol, cholesterol esters, glycerophosphocholine, sphingomyelin) [46]. The variations in lipid species determine EVs physicochemical properties, of which zeta potential is negative due to their membrane negatively charged lipids, such as phosphatidylserine and glycan-moieties [47]. Variation in EVs inter- and intra-vesicles lipid composition depends on the type of generative cell, and the subgroup analyzed [48]. In contrast to LPs, EVs share the lipid asymmetry of the parent cells they are generated from, which is highly beneficial for the interaction with their target cells [49] as such biogenic vesicles can be an approach where hybrid vesicles are designed by fusing LPs and EVs that aid in more asymmetry within LPs lipid bilayer, and hence biocompatibility [50].

### 2.3. Protein Composition

Classically, LPs do not contain any proteins in the lipid bilayer or intraluminal space. However, different studies attempted to design proteoliposomes (proteo-LPs) that incorporate proteins [51,52,53]. Proteo-LPs have been employed as tools for studying lipid-protein and protein-protein interactions as well as topological features of different membrane-associated proteins [54]. While proteins are not generally found in LPs, a variety of proteins have been confirmed bound to EV membranes or present in their intraluminal space. These include heat shock proteins (e.g., Hsp70 and Hsp90), lysosomal-associated membrane proteins (e.g., Lamp2a and Lamp2b), cytoskeletal proteins (e.g., actin, tubulin, and cofilin), major histocompatibility complex class II (MHC class II), endosomal sorting complex required for transport (ESCRT), Alix, tumor susceptibility gene protein 101 (TSG101), integrins, proteoglycans, and tetraspanin proteins (e.g., CD9, CD37, CD53, CD63, and CD81) [55]. Moreover, proteins found within the endoplasmic reticulum, the Golgi apparatus, mitochondria, and some cytoplasmic proteins have been detracted in EVs.

Although engineering LPs play a role in improving their pharmacokinetics and pharmacodynamics as advanced drug delivery entities, bioengineering EVs play a role in embracing therapeutic proteins on their membrane or in their lumen as biocompatible drug delivery vehicles [56]. Protein construction into LPs requires criteria to be considered, such as inserted protein homogeneity, orientation, and morphology, as well as the reconstituted proteo-LPs size and permeability [57]. Proteins can be reconstituted either by detergent-solubilized proteins mixed with an excess of phospholipids and appropriate detergent or by the gradual addition of detergent to dissolve the prepared LPs, followed by the addition of solubilized protein. Finally, the detergent must be removed in order to obtain proteo-LPs [54]. As with lipids and due to the EVs protein sorting mechanisms, the protein composition of the EVs is not identical to that of the parent cells. Thus, understanding EVs protein sorting mechanisms as well as factors such as biogenesis and cell source is essential for controlling cellular protein cargo loading into EVs and protein importance for cellular uptake and pharmacokinetics.

### 2.4. Carbohydrate Composition

The study of carbohydrate composition in EVs came after the other major biomolecules. Carbohydrate structures can be found as conjugated to lipids and proteins as glycans or as repeating glycosaminoglycan chains like in proteoglycans. Glycans are well-known as important structural components in addition to their functions in energy storage. It is estimated that half of all human proteins are glycosylated [58]. However, they were more recently renowned as information carriers and uniquely modulated molecular recognition events at the cellular level. They also function in controlling both intracellular trafficking and quality control of folding events at the level of individual proteins [59,60]. Proteoglycans are the main constituents of the extracellular matrix and are essential to tissue architecture [61]. Aberrant glycosylation disrupts these essential functions facilitating the progression of cancers or resulting in lysosomal storage diseases [62]. Unlike EVs, LPs do not have carbohydrates in their typical structures.

### 2.5. Polymer Composition

Typical LPs are easily taken up and digested by mononuclear phagocyte system (MPS); thus, researchers came out with polymer-grafted long-circulating LPs that have to extend circulation time in vivo. Several polymers were grafted onto the phospholipid structure. Amongst them, polyethylene glycol (PEG) grafted onto PE has received the most attention [63]. PEG is a hydrophilic polymer of ethylene oxide, which is a non-immunogenic, low-toxic, highly biocompatible, and low molecular weight polymer that is tremendously used in biomedical applications [63]. 1,2-distearoyl-sn-glycero-3-phosphoethanolamine-polyethylene glycol (DSPE-PEG2000) is an example of PEGylated phospholipid that serves as a steric barrier to stabilize the molecule assemblies in the LPs. The United States Food and Drug Administration (FDA) approved doxorubicin LPs formulation, Doxil^®^, which contains DSPE-PEG2000 [64].

## 3. Methods of Preparation/Isolation 

LPs are generally formulated from their raw material constituents using different preparation techniques, while EVs are isolated from the biofluids. The methods of LP preparation and EV isolation are explained below.

### 3.1. Liposomes Preparation Techniques

**Figure 4:**
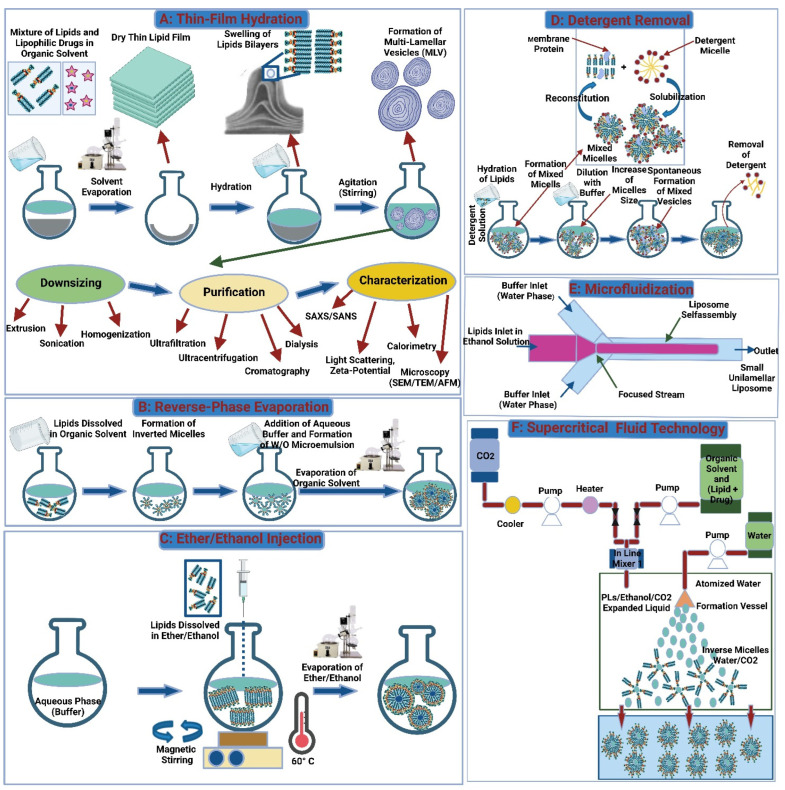
Schematic representation of the main methods of LPs preparation: (**A**) Thin-Film Hydration. (**B**) Reverse-Phase Evaporation. (**C**) Ether/Ethanol Injection. (**D**) Detergent Removal. (**E**) Microfluidization. (**F**) Supercritical Fluid Technology.

LPs can be produced in several ways. Each one affects the LP’s final characteristics, such as size, lamellarity, and drug encapsulation efficiency (the amount of drug successfully incorporated into the LPs) [65,66]. Some approaches used in LP preparation include mechanical process, displacement of organic solvents with aqueous media, removal of detergent, fusion or change in the size of the prepared vesicle, and supercritical fluid technology (Figure 4). More recently, LP preparation has been categorized into conventional methods that are usually performed in the lab and novel approaches that might be used for large-scale production and may require the use of specialized equipment [67,68].

#### 3.1.1. Conventional Methods

##### Thin-Film Hydration

Thin-film hydration is one of the most common mechanical methods for LPs synthesis. The conventional steps for preparing LPs at the laboratory scale begin with the selection of lipids, the formation of combination films consisting of lipids and pharmaceuticals via organic solvent evaporation, low-pressure evaporation to dry the film, and finally, dispersion and homogenization of the film [69]. Bath-type or probe-type sonicators are used to achieve uniform dispersion of vesicles with increased tissue penetration ability [70]. Bath sonication is the preferred sonication procedure due to its simple, gentle, and mild nature, which preserves the sample. On the other hand, the probe sonicators perform better at reducing LPs size. The probe can cause the phospholipid/drug particle to break down and release titanium particles into the dispersion [71]. In general, the thin film hydration method is not preferred in commercial production due to the large amount of organic solvent used during the procedure, resulting in solvent residues present in the LPs, which necessitates complex removal and sterilization steps. As a result, this approach is unsuitable for industrial use.

##### Reverse-Phase Evaporation

Reverse-phase evaporation marked an important phase of LP technology, whereby the synthesis of LPs with a high aqueous space-to-lipid ratio and high entrapment efficiency of aqueous material was introduced [72]. Inverted micelles or water-in-oil emulsions were produced using reverse-phase evaporation. Here, inverted micelles are produced when an aqueous phase containing a drug is added to the organic phase via sonication. By using a rotary evaporator, the organic solvent can be removed under reduced pressure. The dispersion forms a viscous gel, which then turns into a water suspension containing the desired LPs [73]. When compared to thin-film hydration, this approach results in a larger loading of the aqueous component. A disadvantage of this method is that trace amounts of organic solvent may be retained, which can affect the stability of the lipids or encapsulated drugs [74].

##### Ether/Ethanol Injection

In this method, lipids are dissolved in an organic phase, usually ethanol or ether, which is then injected into the aqueous media, resulting in LPs synthesis [75]. Unlike the thin-film hydration method, the use of sonication or extrusion is not applied, which results in small, more stable, single-bilayered LPs with superior dispersal characteristics. The method also declines the use of hazardous materials used in the thin-film hydration process, making it a safer option for workers [76]. The ethanol injection technique is a promising technology for large-scale LPs synthesis applications due to its convenience, quick implementation, reproducibility, and product stability [77,78]. Unlike the ethanol injection method, ether is incompatible with the aqueous phase and requires an additional heating step to remove the solvent from the LPs. In the ether injection procedure, ether-lipid solutions are injected into an aqueous phase at temperatures above the boiling point of ether. Next, the ether is removed over a longer duration, which concentrates the liposomal product and results in a better product with higher encapsulation efficiency [79]. However, this method is unfavorable in some aspects: the resulting liposomal products are heterogenous, and the elevated temperatures used may affect the stability of the encapsulated drug [80].

##### Detergent Removal Method

Detergent removal is a simple, easy-to-implement method that produces a uniform product with precise particle size control. The method starts with dissolving the phospholipids in an aqueous solution. This solution contains detergents at critical micelle concentration (CMC). Individual bilayered phospholipid molecules are produced when the detergent is removed from the reaction medium using a suitable method like a dialysis bag, polystyrene-based absorber beads, or Sephadex columns. Dilution of the resulting mixture with an adequate aqueous medium causes the produced micelles to restructure and evolve into LPs [67,81]. However, this method is not preferable, as it requires a longer duration, retains some of the organic solvent and detergents in the final product, yields low amounts of LPs, has poor encapsulation efficiency, and requires a sterilization step [82].

##### Freeze-Thaw Extrusion Method

Freeze-thaw cycling is a commonly used strategy for reducing the lamellarity of LPs produced, forming a less polydispersed system, and/or disrupting the liposomal bilayer to encourage encapsulation of drug molecules via diffusion [83,84]. Liquid nitrogen (−196 °C) is used to freeze the LPs and then defrost them at a temperature above the lipid phase transition temperature. In literature, freeze-thaw cycles that are required to load the target molecules vary widely, with some articles claiming as many as ten rounds. Multiple freeze-thaw cycles are used to reach drug concentration/solution equilibrium [83,85]. By using this method, larger-sized multilamellar vesicles are produced. Extruding the membrane through polycarbonate filters at desired pore size results in a more uniform LPs distribution with a smaller size [86].

##### Dehydration-Rehydration Method

The dehydration-rehydration approach was developed to achieve high loading rates, especially with labile biological molecules, such as proteins, DNA and RNA. SUV-LPs are synthesized via sonication, then transformed into MLV-LPs. The resulting solution is frozen, then freeze-dried for 16–18 h [87]. The dehydration–rehydration technique provides high drug-encapsulation efficiency and is widely used in nanomedicine [88].

##### Heating Method

In the heating method, PLs are hydrated and heated above the lipid transition temperature in the presence of a hydration agent resulting in the formation of LPs. Some commonly used hydrating agents include propylene glycol, glycerol, and sorbitol. The ability of hydrating agents to exert colonization properties allows the lipid vesicles formed to be more stable by preventing coagulation and precipitation. Also, since these agents are biocompatible, nontoxic, and water-soluble, they do not need to be separated from the final liposomal product [89]. This approach is desirable due to its simplicity, short duration, and removal need for sterilization; however, the high temperatures used can degrade heat-sensitive bioactive molecules [82,90].

#### 3.1.2. Novel Methods

##### Microfluidization

Microfluidization is a technique that uses high-energy mixing of a lipid-containing organic phase with an aqueous phase and passing the liquid through different streams in a channel. The LPs are formed as the organic phase in the lipid stream diffuses and dilutes into the aqueous stream [91]. The mixing ratio, flow rate, and lipid concentration, as well as mixing temperature, can be controlled by the user and result in different-sized particles with varying dispersity [92,93]. This technology is frequently used in industry to produce LPs in continuous processes at large volumes using a microfluidizer [94]. Despite the advantages of this technique in particle size and target molecule loading, the use of organic solvents in the process remains an issue [91,95].

##### Supercritical Fluid Technology

Supercritical fluids (SCFs) exhibit features that are similar to both liquids and gases. They have a solvent power similar to liquids and mass transport properties similar to gases [89]. In LPs manufacturing, SCF methods are thought to be a good alternative to conventional approaches such as thin-film hydration, detergent removal, solvent injection, reverse phase evaporation, and emulsion method [96]. It provides a quick and easy one-step process with improved control of the LP final properties. Furthermore, the use of organic solvents can be lessened or removed [96]. Among the SCFs processes available, supercritical carbon dioxide is the most used for LP production due to its safety (non-toxic, non-flammable), recyclable nature, ease of purification, and stability of the product. The manufacturing process can be controlled by varying the temperature, pressure, and solvents [97].

### 3.2. Isolation of Extracellular Vesicles

**Figure 5:**
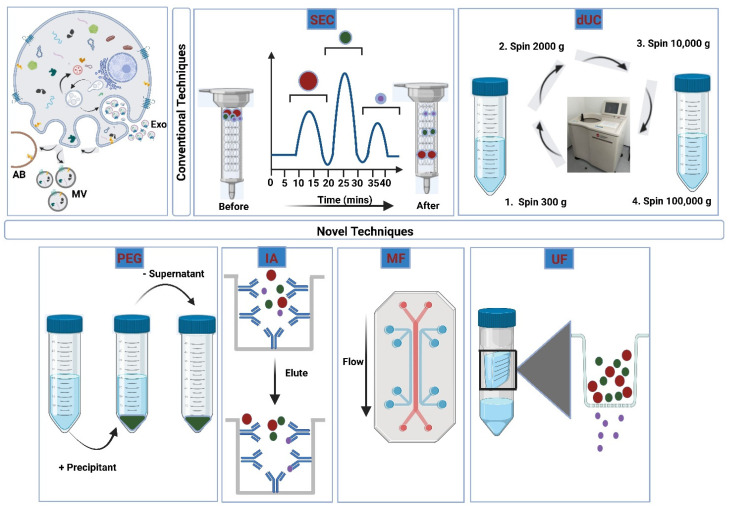
EVs biogenesis-based categorization into; apoptotic bodies (AB), microvesicles (MV), and exosomes (Exo). Methods of EVs isolation include; size-exclusion chromatography (SEC), differential ultracentrifugation (dUC), polyethylene glycol (PEG)-based precipitation, immunoaffinity (IA), microfluidics (MF), and ultrafiltration (UF).

Apoptotic bodies, submicron-sized microvesicles, and nanometer-sized exosomes are EVs released by mammalian cells [98]. Exosomes have gained widespread interest in literature for their potential application in therapeutics for drug loading due to their ability to permeate biological membranes while protecting the drug. Nevertheless, their widespread clinical application is limited by low yields, complex isolation procedures, the need for purification steps, and limited encapsulation. Modification of the innate properties of the EVs, along with the properties of the drug, is necessary before they can be efficiently used in clinical therapy [99]. The first step in EV manipulation is extraction from human serum, followed by isolation from protein and lipoprotein impurities [100]. The challenge in isolating and purifying EVs is due to the viscosity of the human serum and the presence of non-EV proteins and lipid particles in the serum [101]. On top of that, different samples require different isolation and purification steps due to the nature of the EV origin, which can be either sourced from cell culture media or from body fluids [102]. The separation protocol is chosen depending on the origin of the sample, the type of study (basic vs. clinical), the research objectives, the need for scale-up and reproducibility, and the final application of the EVs [100]. In EVs destined for clinical application, it is important to standardize the isolation method to be able to achieve similar yields and purity in the final product. Ultracentrifugation is the standard gold method for the separation of exosomes. However, to address the limitations of ultracentrifugation, alternative methods were developed, including size sorting, immunoaffinity, and sedimentation of the exosomes [35,103] (Figure 5).

#### 3.2.1. Ultracentrifugation

EV isolation is performed via a combination of differential centrifugation with ultracentrifugation; in this process, the sample is centrifuged serially to remove cellular debris, and a final ultracentrifugation step is done to separate the EVs in a pellet [104,105]. The exact parameters of the method used, i.e., speed, number of washes, centrifugation time, and clearing factors, are chosen based on the sample origin, and that influences the quantity and quality of the separated EVs [106,107]. The maximum speed of the equipment combined with the viscosity of the sample is important consideration points, as they determine the k-factor and efficiency of the separation, respectively [108]. Ultracentrifugation is a preferred method due to its economic cost, large volume processing, high yields, and lack of the need for chemicals [105,109]. However, the process can be laborious, requiring a long centrifugation time and poorly scalable, while the EVs isolated are of poor yield, poor purity, and of lower quality due to aggregation of the EV and co-sedimentation with other particles, which limits its effectiveness [110,111]. The use of a sucrose gradient coupled with careful design of the centrifugation and ultracentrifugation steps to remove the impurities and isolate EVs stepwise can help to overcome these limitations [112].

#### 3.2.2. Size-Exclusion Chromatography

The size-exclusion chromatography (SEC) is a standard method for the separation of large molecules from various samples, including EVs from cell culture samples and human blood samples [113]. The principle of the method is molecular size and hydrodynamic volume-based separation of the target molecule (in this case, EVs) from other components. The SEC setup has two parts: a chromatographic separation column (stationary phase) and a pump to aid in elution. For the isolation of EVs, Sepharose CL-2B is frequently used as a stationary phase which produces EVs with high yield and purity [114]. In order to perform the separation; the samples are first concentrated or pre-filtered to remove contaminants before being introduced into the separation column [115]. SEC is routinely used to separate larger varieties of EVs but separating discrete EVs remains a difficulty due to the technique’s poor resolution. As a result, more than 40% of research has coupled SEC with various strategies to overcome SEC restrictions and increase EV purity [113].

#### 3.2.3. Ultrafiltration

Ultrafiltration relies on the use of membranes with pre-determined pore sizes to separate particles of a specific size range [116]. In the ordinary filtration process, the target solution is allowed to flow continuously through the membrane. As a result, targeted particles accumulate in the filter, which reduces the number of open pores available for the flow of the solution and causes lower success of the filtration process [117]. In order to overcome this limitation; tangential flow filtration (TFF) was introduced [118,119]. Traditional TFF systems rely on a single separation unit with one filter membrane. Thus, it is important to extract and concentrate EVs from a small volume of samples to be able to apply the TFF system for therapeutic molecule purposes [120]. More complex platforms that employ dual-filtration techniques and cyclic TFF systems have been introduced in recent investigations for high-throughput applications [121]. Despite the rapid introduction of technologies to improve filtration output, some limitations remain, including the low concentration of EVs, separation of proteins alongside target EVs, poor purity of filtered EVs, and filter clogging with EVs. As a result, isolation processes should be modified for maximum EV recovery and higher concentration and purity [122].

#### 3.2.4. Polymeric Precipitation

The polymer-based precipitation approach is another method for EV isolation and biomarker assessment. Commercialization kits and PEG precipitation fall under this approach [123]. EVs are poorly soluble in PEG solution. Hence this principle is used in commercial kits for EV isolation [124]. PEG is a synthetic polymer with high water solubility, cheap, effective, non-toxic, and non-denaturing, making it an excellent polymer choice for the precipitation of EV. Combining PEG with dextran resulted in two-phase separation with improved purity from other proteins [125]. The PEG acts as a polymer mesh selecting EVs at a certain size range, which can later be separated further using centrifugation [102]. This method is rapid and effective for the separation of EVs from different origins and does not require repeated centrifugation cycles [126]. Nevertheless, the drawbacks of this method include poor purity values, which affect the final EV function and quality [127].

#### 3.2.5. Immunoaffinity Isolation

Immunoaffinity refers to the affinity of an antibody to an antigen [128]. EVs isolation using immunoaffinity can be achieved via various means. In one way, the starting sample is mixed with inert materials, like magnetic beads [129] or gold-loaded ferric oxide nanocubes [130], coated with antibodies against the antigens. In another way, the sample can be mixed with surface receptors from parent cells, such as chondroitin sulphate peptidoglycan 4 [131], epithelial cell adhesion molecule [132], or exosome-binding molecules like heat shock protein [133] and heparin [134]. Immunoaffinity is frequently employed in conjunction with differential ultracentrifugation to purify the isolated EVs. While being effective, this method is limited by the types of markers that selectively isolates certain EVs. Although this results in lower yields, the purity achieved is higher due to high selectivity. Another limitation of this method is the additional processing step for the removal of the antibodies from the vesicle surface, which can challenge the integrity of the EVs [135]. Lastly, most commercial antibodies are of a limited type, which cuts off the specificity and quality of the target EV. The use of immunoaffinity methods for large-scale commercial EV isolation is impractical because it is expensive to scale up and the high price of highly specific antibody-conjugated molecules. Hence, the application of immunoaffinity isolation of EVs is limited to the research laboratory [136].

#### 3.2.6. Microfluidic Devices 

The action of microfluidic instruments for EV separation can be categorized as either “size-based” or “immunoaffinity-based” [137]. Microfluidic approaches have been proven to provide a precise and sensitive classification of various EV types while minimizing reagent use, production cost, and process duration [108]. EV isolation using this method is useful for the selection of EVs with specific antigens, sizes, and densities. In microfluidics-based immunoaffinity capture techniques, the cell culture medium of human blood is sampled to isolate EV targets with specific antigens [138]. The EVs are selected using capture antibodies or beads coated with capture antibodies for the surface antigen [139]. Meanwhile, microfluidics-based membrane filtration makes use of the size of the EVs and can be pressure or electrophoresis driven. Here, a porous membrane with nano-sized pores is used to select the target EVs [140]. Using this method, up to 4 filtrates can be collected before the pores become clogged. The addition of an electrophoresis step removes the clogging and improves the efficiency of microfluidics-based membrane filtration and the purity of the collected EVs [140]. The nanoscale deterministic lateral displacement (nano-DLD) is a pillar-array-based microfluidic device that selects particles flowing continuously [138], while nanowire-based traps (NTs) use nano-sized wires to filter particles of the desired size. Viscoelastic flow systems use viscoelastic forces to isolate EVs from the starting cell media or blood [141]. The isolation process is based on size and follows a continuous flow to produce very high-purity EVs with good yields [141]. In the acoustic system for separating EVs, nano-sized particles are isolated based on their physical properties, such as flexibility, size, and density [142]. The use of acoustic waves has also pushed boundaries in micro separation technology. The new technology offers easy fabrication of components and compatibility with other microfluidic parts, non-immunogenicity, and fully automated nanoparticle manipulation [143].

## 4. Drug Loading into Liposomes and Extracellular Vesicles 

### 4.1. Drug Loading into Liposomes

LPs are routinely used in the delivery of therapeutics. Thus, high drug loading in the vesicle is an important consideration in the production of LPs for therapeutic applications, in addition to reducing the number of vesicles delivered per dose. The method used in drug loading should not only ensure high encapsulation efficiency for the final product but also reduce the cost of drug loss from poor uptake into the LPs [144]. LPs are effective drug delivery vehicles for compounds with different water solubilities, where the drug molecule is loaded in various parts of the LP. Loading of LP usually follows the thin-film hydration technique; this method favors the loading of lipophilic drugs due to high uptake in the lipid bilayer. However, this technique is less suitable for encapsulating hydrophilic drugs due to the limited uptake area in the core of the LPs. Hence, it is important to decide the best LPs synthesis method for encapsulating drugs with different physicochemical properties and using different starting lipids [67]. Drug loading can be achieved via passive or active loading methods. The choice of method can affect the final LPs quality and quantity of the loaded drug, as well as the end properties of the LPs [145]. The main deciding factor for effective drug loading is the property of the drug being encapsulated [69]. Drug loading into LPs is commonly performed during the fabrication process, but it can also be achieved post-fabrication. The latter method relies on preparing blank LPs and then loading the drug into/onto them. Below are different approaches to LPs drug loading in more detail. 

#### 4.1.1. Passive Loading Approach

In passive loading, the drug is loaded before or during the preparation of the LP [146]. Osmosis takes place between the drug in the solution and inside the LP until an equilibrium is reached. The excess drug in the solution is washed off after loading is complete. This technique is suitable for loading lipophilic or amphiphilic compounds, which can partition in the LP lipid bilayer and the aqueous solution [144]. This approach, however, sometimes results in poor loading efficiency [147]. Drug solubility, LP-size, lipid concentration, and method used for synthesis all affect the efficacy of passive loading [148]. The average drug-to-lipid ratio attained by this strategy is less than 0.05 (*w*/*w*) [149,150]. Furthermore, because of the weak interaction between the drug and the LPs, the entrapped drugs are not stably loaded, resulting in poor stability of the LPs and retention of the loaded drug. LPs loaded via this method commonly undergo a “burst release” pattern, where the loaded drug is rapidly released in large amounts [148].

#### 4.1.2. Active Loading Approach

In addition to loading drugs during the synthesis of LPs, drugs can also be loaded post-formation of the LPs. This loading process, also known as active loading [146], is made possible by the flexibility of the LP. Since the drug is loaded after LP synthesis, there is less damage to the biologically active molecule from the LP synthesis step [145]. Compared to passive loading, active loading results in increased efficiency and loading of the drug, higher drug retainability, and better drug stability during storage. After the production of intact vesicles, chemicals can be delivered into the LPs using transmembrane gradients, such as electrical, ionic, or chemical potential gradients. An active loading technique known as remote loading has been used to load the amphipathic drug doxorubicin into LPs. Here, the drug permeates the LP bilayer and is trapped in the LP core, where it is held securely, resulting in a drug vehicle with better pharmacokinetic and safety profiles [148,151].

### 4.2. Drug Loading into Extracellular Vesicles 

The finding that functional RNA and protein can be carried via EVs into cells has led scientists to focus on the principles that surround drug loading into EVs and their delivery to target cells [152]. The use of EVs for the delivery of therapeutics is advantageous, as the lipid bilayer membrane protects the drug loaded in the EV cargo from being damaged once introduced into the cells. Ironically, this layer also complicates the loading of the drugs [153]. The introduction of the drug into the EV can be via cell-based (also known as endogenous loading) or non-cell-based (also known as exogenous loading) approaches; both have applications with different drugs and their own pros and cons [154].

#### 4.2.1. Cell-Based Loading Approach

Cell-based loading, i.e., endogenous loading, refers to drug loading via EVs through cell transfection with expression vectors. In this approach, the cells are bioengineered to load the active ingredients into EVs. The cell is manipulated into producing the desired drug, commonly biopharmaceutical. Delivery of the resulting EV into the target cell is achieved by attaching specific receptors to the EV surface [155]. In order to load the drug into the donor cell, transfection and incubation are common strategies used. In transfection, the oligonucleotide sequence or plasmid is introduced into the cells to induce the cells to express miRNA/siRNA/mRNA. Common transfection procedures include the calcium phosphate method or they can be lipid-assisted using Lipofectamine, HiPerFect, or Exofect transfection agents [155]. Endogenous loading using cells allows the process to be scaled up and the drug directly loaded into the EV; however, the loading efficiency can be poor [156].

#### 4.2.2. Non-Cell-Based Loading Approach

Exogenous EV loading methods, also known as the non-cell-based EV loading approach, refer to the introduction of drugs into EVs after their isolation. Exogenous loading can be classified into two types: passive and active [157]. In passive loading, the EVs are mixed with the target molecule over a certain duration to allow passive uptake of the drug into the EVs [156]. The process is straightforward and does not require the use of external stimuli. However, the time of co-incubation will depend on the physicochemical properties of the loaded drug [158]. Hydrophilic and neutrally-charged molecules use osmosis to diffuse through the bilayer membrane, whereas hydrophobic molecules use hydrophobic interactions to integrate with the lipid membrane [159]. Passive loading is frequently associated with poor encapsulation efficiency and reduced accuracy in the determination of the final loaded vesicle purity. In studies investigating the encapsulation of paclitaxel, Agrawal et al., 2017, reported 8% loading in milk-derived EVs after 15 min [160], while Kim et al., 2018, reported 1.4% loading in macrophage-derived EVs after 1 h [161]. While these studies provide comparisons for the loading of drugs, careful consideration of the differences in methodologies needs to be taken; some studies report that alteration of the target drug’s physicochemical properties has increased the efficiency of passive loading. A hydrophobic moiety, for example, can be added to siRNA to improve its loading, with up to 80% efficiency achieved with this simple addition [131,162].

The active loading of EVs necessitates some form of EV membrane rupture [156]. Active transfection processes such as sonication, extrusion, freezing and thawing cycles, saponin, dialysis, electroporation, and chemical-based methods use physical ways, electric current, or detergent to disrupt the EVs membrane and increase permeability to achieve encapsulation of the target molecule at high efficiencies [159,163]. Sonication employs sonicator equipment to apply sound energy to an EV solution. Mechanical shear is produced, which impacts the integrity of the EVs membrane, and enhances the uptake of loading material into the vesicles [164]. Meanwhile, in extrusion-based loading, extruded EVs pass through small polycarbonate porous membranes, allowing target molecules present in the solution to be incorporated into the vesicles [165]. Another technique for encapsulation, known as the freeze-thawing cycle approach, entails freezing and then thawing a solution of vesicles containing the desired molecules thrice to induce uptake of the molecule [166,167]. The use of saponin to permeabilize the EVs membrane is another approach for drug loading. Saponin acts as a detergent, removing cholesterol to produce pores in the membrane while preserving the rest of the membrane structure. This method is employed to encapsulate dyes and molecules that are otherwise not able to penetrate the membrane. For the loading of therapeutic drugs, hypotonic dialysis is a preferred method due to its use of gentle osmosis mechanisms for loading. The EVs are disseminated in a hypotonic solution, where they then expand and form pores. This formation of these pores allows soluble drugs to penetrate the membrane and load into the vesicle. The integrity of the EV is returned when the filled vesicles are placed into an isotonic solution, resulting in the final loaded product [168].

The application of an electrical field to promote the permeabilization of the EV membrane is known as electroporation [169]. It is one of the most common techniques to improve encapsulation [170]. Fuhrmann et al., 2015, compared various engineering methods for encapsulating porphyrins into EVs of various biological origins. The authors reported electroporation as the best method for increasing encapsulation efficiency [171]. In addition to using electrical pulses via electroporation, transfection can also be induced via chemical means. Using chemicals assists with high transfection success rates in vitro; however, transfection success is higher with electroporation. The use of chemicals for transfection has its limitations, as it is undesirable for producing particles for use in therapy due to the toxicity of the chemicals used, incomplete loading capacity, and difficulty differentiating EVs and derivate reagent aggregations [167]. When compared to endogenous loading, active loading strategies such as electroporation and sonication have the advantage of higher loading efficiency. Nonetheless, some limitations of these techniques have been reported in the literature, such as substantial aggregation of RNA-loaded material, changes in EV shape, and DNA/RNA degradation [172]. Furthermore, additional stages are required in the manufacturing process, and the use of expensive chemically modified oligonucleotides in the case of nucleic acid delivery becomes necessary [156]. The uptake of nucleic acid cargo can be improved by creating a pH gradient between EV membranes [172]; this does not create an energy output that may damage sensitive DNA/RNA targets, which is associated with sonication and electroporation methods.

## 5. Pulmonary Drug Delivery

### 5.1. Overview of Pulmonary Drug Delivery

**Figure 6:**
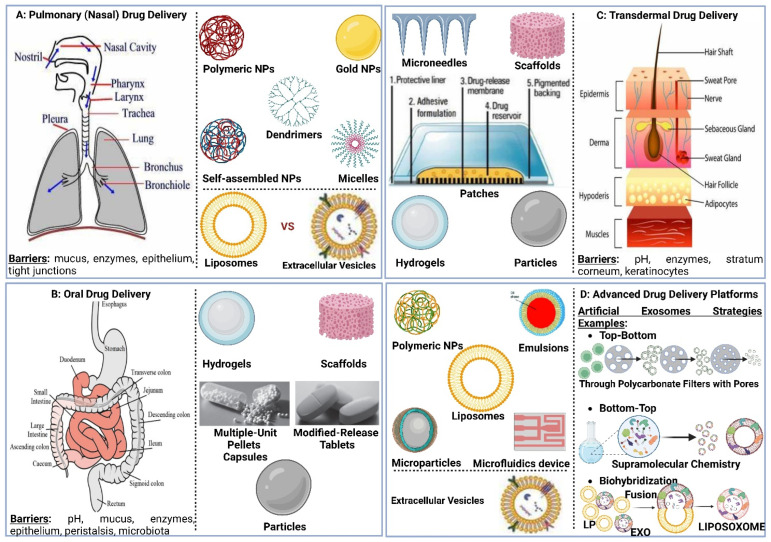
Advanced drug delivery platform technologies to improve the route of administration compliance: (**A**) Pulmonary drug delivery platforms. (**B**) Oral drug delivery platforms. (**C**) Transdermal drug delivery platforms. (**D**) Advanced drug delivery platforms.

Pulmonary drug delivery (PDD), in comparison to many traditional routes of administration, offers several advantages for systemic and local treatments [173]. These advantages include the lung’s large surface area, high blood perfusion, and bypassing of the first-pass metabolism in the liver [174]. As a patient-friendly route of administration, the lungs are a promising portal for biopharmaceuticals delivery (e.g., protein therapeutics and vaccines) as well as bioengineered EVs (BioEVs) (i.e., futuristic biopharmaceuticals) that are typically administered parenterally [175]. Inhalation is the preferred method for treating respiratory diseases, as it targets the site of action and thus rapid onset, is painless and thus improves compliance, and evades first-pass metabolism and thus minimizing systemic side effects [176]. In contrast, utilizing the lungs as a gateway for systemic drug delivery, drug molecules’ access into the systemic circulation beyond their respiratory deposition and dissolution remains size-dependent for transportation across the respiratory epithelium [177]. Figure 6 summarizes the advanced drug delivery platform technologies developed to overcome barriers associated with PDD compared to other administration routes.

Despite the major advantages of PDD over intravenous (IV) drug administration, inhaled therapy is limited by several dams to reach an effective therapeutic dose useful in the treatment of respiratory and/or systemic diseases. Without an optimal inhaler, a successful drug inhaler formula will not achieve its target delivery goal. Various inhalers can be used to administer orally inhaled drug products to patients, including DPIs, pMDIs, and nebulizers [178,179]. The success of LPs as inhalable carriers leads to more focus on research and development in the design of effective aerosolization devices as well as LP formulations for local and systemic targeting. Also, the small size of the LPs (average ≈ 100 nm) enhances their permeation and retention (EPR) within the respiratory tract and controlled release of the encapsulated drug for local targeting as well as systemic circulation [180]. Regardless of EVs composition complexity, EV’s small size (exosomes average ≈ 110 nm) confers the same properties as LPs concerning EPR, whereas EVs traverse biological barriers via receptor-mediated transcytosis property as an extra bonus [181]. Three barriers hinder aerosol deposition deep in the lung (bronchioles and alveoli), are: (I) anatomically—the structure of the tracheobronchial tree, (II) pathologically—disease status of the respiratory system, and (III) immunologically—alveolar macrophages and mucociliary escalator [182]. Furthermore, inhaled drug carrier fine particles fraction (FPF), to be therapeutically useful, should have a mass-median aerodynamic diameter (MMAD) smaller than 5 µm for bronchioles deposition with respect to local targeting and less than 2 µm for alveolar region deposition with respect to systemic targeting [183]. Hence, the prime factor in overcoming these barriers is the size of inhaled particles. Accordingly, LPs and EVs, as well as BioEVs, can be aerosolized into particles of high FPF, where LPs and EVs encapsulate/load a drug concentration that is therapeutically feasible, whereas BioEVs are considered pharmacologically active entities.

To formulate LPs and EVs as effective inhalation therapies, respiratory anatomy, inhaled particles mechanisms of deposition, and mechanisms of respiratory defense should be fully reviewed. The respiratory tract is subdivided into conducting part, which consists of the nose, nasal cavity, and pharynx, and the respiratory part, which consists of the larynx, trachea, bronchi, alveoli, and lungs. Lungs are subdivided into five lobes. Where the right lung contains three lobes, while the left lung contains two lobes.

**Figure 7:**
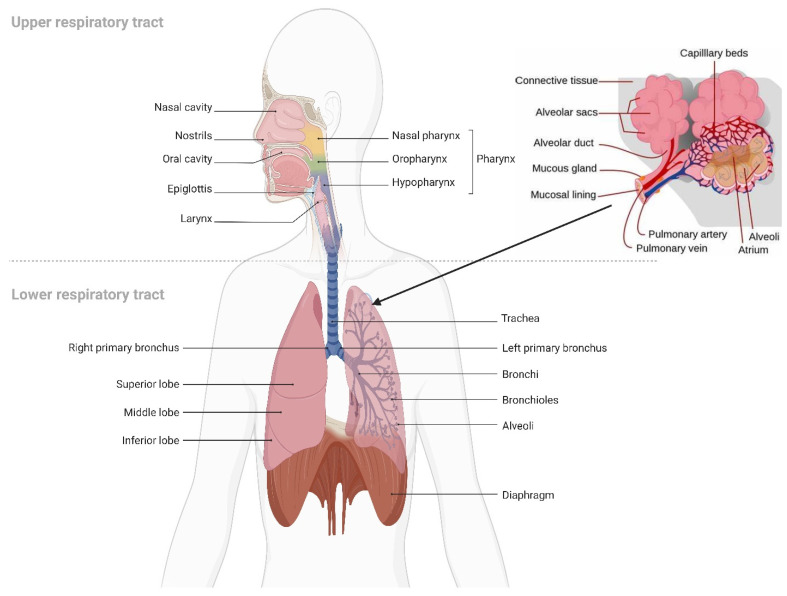
Human upper and lower respiratory tracts.

The bronchi are subdivided into more narrow bronchioles that conduct air and end with the alveoli, where gas exchange takes place [184]. Each lung contains about 300 million alveoli, which are lined with pulmonary capillaries totaling over 280 billion capillaries, forming a massive network which provides a surface area of about 70 m^2^ available as a blood gas barrier. Alveolar gas exchange occurs by diffusion mainly at the interface between capillaries and alveoli with a distance as small as 0.5 µm consisting of the alveolar epithelium, endothelium, and interstitial cell layers [184] (Figure 7). The uniqueness of the lung anatomy is very attractive for targeting drug delivery due to its large absorptive surface area and high blood perfusion, constituting a non-invasive route for drug administration.

### 5.2. Requirements for Pulmonary Drug Deep Deposition 

Anatomically and functionally, the respiratory system is specially designed so that air in the cleanest possible condition can reach the deepest regions of the lungs. Such an efficient system prevents certain particles from entering the lungs, the same thing used on purpose to deposit drugs into the airways and even into the alveoli [185]. Achieving such a goal requires respiratory tract anatomy, function, and physics laws understanding that govern the design and development of both inhaled drug formulation and inhaler devices.

#### 5.2.1. Inhaled Drug Formulation

The main obstacle for an inhalation therapy formulation that has a high therapeutic activity, and a prolonged release characteristic is the low pulmonary drug bioavailability. This is due to the efficient respiratory clearance mechanisms and immediate systemic absorption [186]. Since the pulmonary bioavailability of a drug is the main parameter of typical therapy, many approaches have been adopted to delay drug absorption and prolong the drug’s pulmonary retention and half-life. The most useful approach was to load the inhaled drug into particulate-based delivery carriers. This approach has many advantages, such as; preventing drug enzymatic degradation, evading respiratory tract clearance, slowing drug absorption, pulmonary drug targeting, controlling drug release, reducing dose frequency, maximizing therapeutic activity, and minimizing side effects [187]. In order to achieve an effective particulate-based PDD system, several factors should be tailored and optimized. These are as the followings:

##### Particle Size and Deposition Pattern

**Figure 8:**
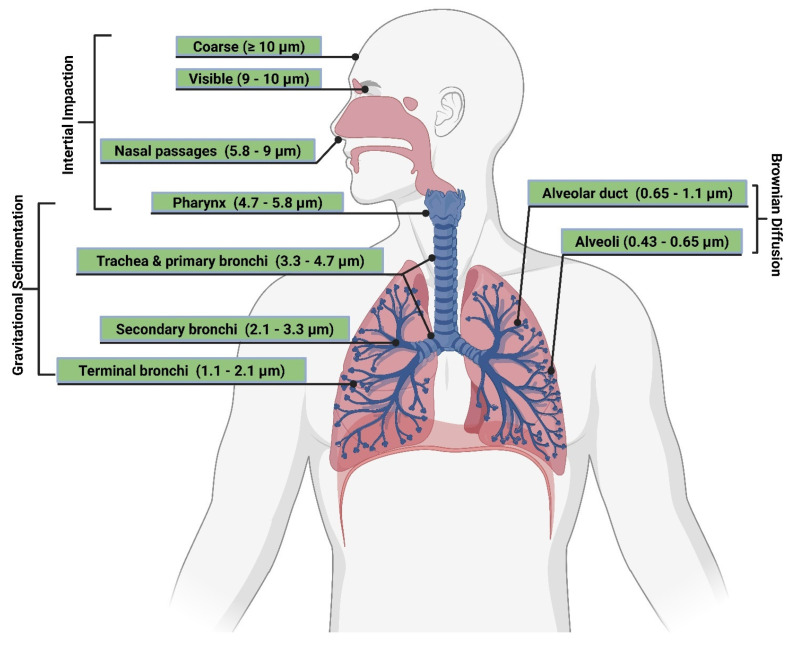
Inhaled particle sizes and respiratory deposition patterns.

The size of inhaled aerosol particles plays a prominent role in terms of drug deposition and pulmonary bioavailability, which, by well-modulating, can target the drug to the site of action and evade respiratory clearance [188] (Figure 8). The extent of pulmonary deposition depends on the patient’s physiology, including breathing patterns and the health status of the lungs, and the physical and chemical properties of inhaled particles, including size, shape, bulk density, hygroscopicity, and moisture content [189]. Mechanisms controlling aerosol deposition mainly include inertial impaction, gravitational sedimentation, Brownian diffusion, and to a lesser extent, interception and electrostatic precipitation [190]. The common deposition mechanism for DPIs and pMDIs is inertial impaction, in which particles of MMAD larger than 5 µm are deposited in the bronchial regions. In contrast, particles of MMAD between 1–5 µm are significantly settled out in smaller bronchioles and alveoli by gravitational sedimentation where airflow velocity is low. Furthermore, particles less than 1 µm in size are deposited in the deep regions of the alveoli via Brownian diffusion, while particles less than 0.5 µm are exhaled during exhalation [191].

**Figure 9:**
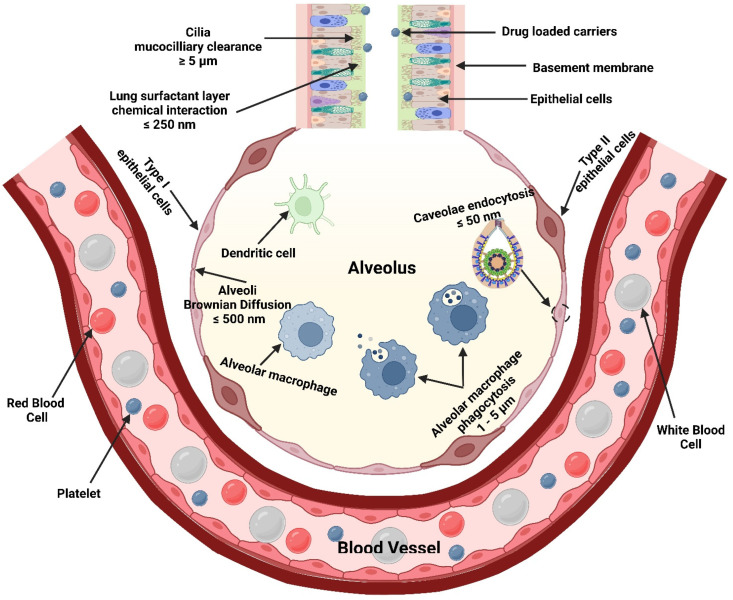
Inhaled particle sizes have different routes for distribution, clearance, and absorption.

Mucociliary escalator and alveoli macrophages are the main causes of therapeutics inhalation failure, which is exacerbated during respiratory diseases. Although particles of MMAD between 1–5 µm can evade the mucociliary escalator and deposit deep into the respiratory tract, they are rapidly engulfed by alveoli macrophages [192] (Figure 9). In order to circumvent the effect of alveoli macrophages, specific particulate-based drug delivery systems based on MMAD modulation can sustain drug release deep into the respiratory tract. A viable approach is the use of large porous microparticles (LPMPs) larger than 5 µm and a density less than 0.1 gL^−1^, which are able to evade macrophage uptake and deposit homogenously deep into the respiratory tract [193]. In contrast, nanoparticles (NPs) appear to be useful for pulmonary delivery but are mostly exhaled after inhalation. In order to solve such a problem, a novel particulate-based drug delivery system was established based on the encapsulation of drug-loaded NPs into microparticles, known as “Trojan,” particles, which showed the ability to deliver NPs into the peripheral airways, thereby avoiding the mucociliary escalator and prolonged drug release. Spray drying of NPs is typical for the production of Trojan particles, followed by their aggregation into less than 0.1 gL^−1^ dense hollow microparticles [194].

**Figure 10:**
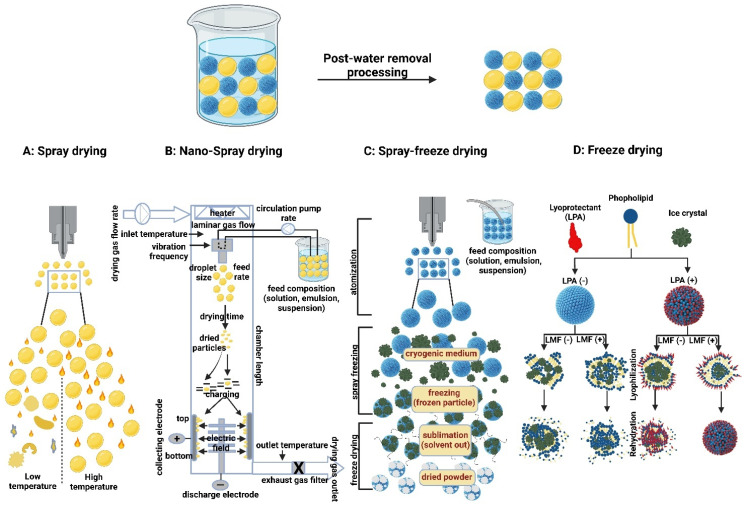
Post-water removal processing technologies for lipid bilayer vesicles (LPs and EVs): (**A**) Spray drying technology. (**B**) Nano-Spray Dryer B-90 HP (Büchi Labortechnik AG Switzerland) technology. (**C**) Spray-freeze drying technology. (**D**) Freeze-drying technology.

Similarly, spray drying of LPs has been shown to be typical for the production of small MMAD particles, i.e., high FPF, as they will be reconstituted after the deposition of the powder in the aqueous environment of the lung [195]. Spray drying is more applicable for the production of various inhalation therapeutics, such as vaccines, peptides, and proteins. It eliminates the need for pricey tools and prolonged multi-step processes because it is a one-step procedure. Additionally, it can modify the end product particle size by adjusting the spray droplet size and solute concentration. This makes spray drying different from lyophilization, which requires mechanical grinding in order to reduce particle size [196]. Spray drying can be used on EV formulations in a similar way. The process of spray drying begins with atomizing the solution containing the EVs so that the droplets can be quickly transformed into a dry powder by using hot gas. As a plasticizer lowers the solid-state glass transition temperature and improves chemical stability, residual moisture makes EVs more stable. The stability and payload of EVs can be significantly impacted by crucial variables such as the rate at which EV solution is introduced into the system, atomization pressure, and outlet temperature. Because of this, these crucial process parameters need to be defined and kept within a small window. A method to encapsulate the platelet-rich EV solution as a potential candidate for wound healing was patented by Behfar et al. in 2016 (US patent number: US20160324794A1) [197]. Fortunately, these variables can be controlled more effectively using nano-spray drying technology (e.g., Büchi Nano Spray Dryer B-90 HP), where the range of powder particles produced extends to the sub-micron- and nano-scale with very narrow size distributions and sample quantities in the milligram scale with high throughput [198]. In contrast, in order to achieve lyophilized intact lipid nanovesicles (i.e., LPs and EVs), an advanced lyophilization technology has been developed that combines liquid-mediated freezing (LMF), e.g., isopropanol was optimal, and lyoprotectant, e.g., trehalose for internal- and sucrose for an external-aqueous phase in order to retain the lipid nanovesicles integrity. During the freezing step, the lyoprotectant prevents supercooling of the water, while LMF controls the freezing rate, which is a critical factor for membrane disruption [199] (Figure 10). However, further investigation is needed to apply these techniques on a large scale to manufacture and store EV-based therapeutics.

##### Particle Shape and Surface Morphology

The primary factor influencing particle aerosolization and pulmonary deposition is particle size, followed by particle shape and surface appearance [200]. This is mainly by affecting the clearance of alveolar macrophage, where the shape and orientation of the particles greatly influence phagocytosis. When compared to non-lipid-based carriers, lipid-based nanocarriers (such as LPs and EVs) often demonstrated considerably greater accumulation and longer retention in the lungs following inhalation treatment [201]. In order to reduce the exhaled tendency of NPs after inhalation, they are formulated as microparticulate-based delivery systems (e.g., Trojan particles) with different shapes and surface morphologies.

Phagocytosis initiates in at least one orientation for all shapes (i.e., elliptical disks, spherical, oblate ellipsoids, rectangular disks, and worm-like shapes). The engulfment is accelerated in less than 6 min by the macrophages’ adhesion to the major axis of elliptical discs. The spherical particles, regardless of where they were attached, were engulfed instantly. However, attachment of macrophages to the flat surfaces or minor axes of rectangular, elliptical disks, and oblate ellipsoids, are failed to engulf even after 2 h. Additionally, the worm-like particles had a considerably lower rate of phagocytosis clearance than the spherical particles because of their reduced bending area [202]. Therefore, in order to prolong lipid-based nanocarriers’ pulmonary retention and delivery periods, particle-engineering technologies are employed to embed these NPs in MPs of different morphologies.

##### Particle Hygroscopicity

A solid’s capacity to absorb moisture until it achieves equilibrium with its surroundings is known as hygroscopicity. Moisture absorption depends on the surrounding relative humidity as well as solid particle core/coat hydrophilicity [203]. The phenomenon of moisture absorption from the surrounding alters many features of the particles, e.g., the bulk density, the surface charge, and the aerodynamic size [204]. Zhou et al., 2013, have shown that spray-drying of colistin powders highly absorbs moisture up to 30%, where the FPF decreased from 80% to 63.2% when stored at 60% relative humidity [205]. Hybrid polymer/lipid anisotropic “Janus NPs” were manufactured using mixtures of biodegradable and biocompatible substances. They have two distinct phases– a polymeric polar phase that can be loaded with hydrophilic drugs and a lipid non-polar phase that can be loaded with lipophilic drugs. During nebulization, Janus NPs can efficiently maintain their shape, size distribution, and drug loading [205]. Schattling et al., 2015, fabricated Janus sub-compartmentalized assemblies (microreactors) with enzyme-loaded LPs (Janus capsosomes) entrapped within a hydrogel carrier shell. This is a surprising step towards artificial polar cells via assemblies with spatial control over liposomal subunit position [206]. Functionally, EVs have some general features of “Janus face” due to their complex biology in both generating and uptaking cells [207]. Hence, fabricating BioEVs loaded Janus microreactors will serve their biological potentials concerning their cellular uptake and biological barrier evasion.

##### Particle Surface Charge 

The development of particle surface charges arises from factors that directly affect particle aerosolization. Particle size or aerodynamic diameter and surface characteristics, such as crystal lattice structures, surface energy, and surface area, influence particle surface charges [208]. Surface charge, particle size, and preparation process are among the most notable characteristics that are influenced by lipid combination in the production of LPs, which are usually made from natural or synthesized phospholipids. The surface charge of LPs can be positive, negative, or neutral due to functional groups on the surface of the LPs at the environmental pH. The cholesterol content of LPs can vary and adapt to the phospholipid packing, membrane fluidity, and surface charge, which impacts particle size, encapsulation effectiveness, and final morphology [145]. Smaller particles have a more active surface area, which increases the cohesion interparticularly and with the surface wall of the inhaler and hence reduces the FPF, based on the relationship between particle size and net surface charge [209]. Similar relationships exist between particle shape and net surface charge, with sphere particles being less likely than elongated particles to pick up charge. Surface charges can also affect how the lungs retain and clear inhaled particles, e.g., cationic NPs were rapidly absorbed by pulmonary epithelial cells and macrophages after administration compared to neutral and anionic NPs of similar hydrodynamic diameters [210]. Due to the increased inter-particle and particle-surface contact area, particles with rough surfaces have a strong tendency to exchange charges [211]. Particulate-based proliposomes (pro-LPs) electrostatic charges influence aerosolization during inhalation, which is further transmitted to the drugs [212]. As EVs are biological lipid bilayer vesicles, their surfaces are negatively charged due to enrichment with phosphatidylserine [213]. Inertial impaction, gravitational sedimentation, and Brownian diffusion are generally responsible for the patterns of particle deposition in the peripheral and deep respiratory regions. In contrast, electrostatic charges play a role in particle deposition via cohesive attraction more in the deep respiratory regions [214]. Thus, optimizing particle surface charge during formulation is highly important to achieve the required particle deposition pattern within the respiratory tract.

#### 5.2.2. Inhalation Drug Delivery Devices

**Figure 11:**
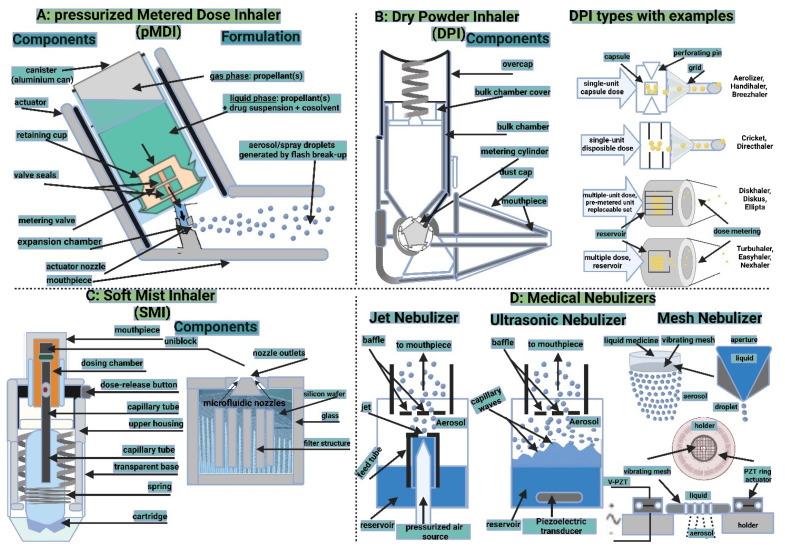
Schematic representation of inhalation drug delivery devices: (**A**) pressurized Metered-Dose Inhaler (pMDI). (**B**) Dry Powder Inhaler (DPI). (**C**) Soft Mist Inhaler (SMI). (**D**) Medical (jet, ultrasonic, and mesh) nebulizers.

There are four main categories of technologies that govern inhaled product delivery: pressurized metered-dose inhalers (pMDIs), dry powder inhalers (DPIs), soft mist inhalers (SMIs), and medical nebulizers (Figure 11). Each of these technologies has undergone testing for the delivery of LPs [215]. The roles of EVs in the mitigation or exacerbation of pulmonary diseases via intercellular cross-talking are well documented, and they exhibit promise for use as therapeutic agents and as biomarkers in diagnostics [216]. The smaller size of EVs eases their deposition in smaller airways and alveolar regions, just like LPs, where EVs’ better-architectured lipid bilayer improves their stability in tissues and body fluids. In contrast to cell therapies, EVs show lower levels of immunogenicity and toxicity [217]. Hence, regardless of pulmonary delivery, whether targeting locally or systemically, EVs can comply with all inhalation devices.

##### Pressurized Metered-Dose Inhalers

Pressurized metered-dose inhalers (pMDIs) are sturdy containers that hold medication dissolved or dispersed in liquefied propellants. Device actuation–patient inspiration, i.e., device–patient coordination results in precise inhalation dosimetry [218]. The propellants rapidly evaporate due to their high vapor pressure, allowing the patient to breathe in the aerosolized medication particles. The pMDI has been the most popular aerosol generator prescribed for patients with asthma and COPD since its creation by Dr. George Maison in 1955. However, concerns have been expressed about their usage in both clinical and environmental settings due to the low dose that reaches the deep lung and the possible ozone layer depletion caused by chlorofluorocarbon (CFC) propellants [219]. According to reports, pMDIs are used to deliver LPs by dissolving the phospholipid in CFC propellant that contains medicines and cosolvents. When the device is actuated, a drug and lipid combination is deposited in front of the impinger, where it is then hydrated to form LPs inside the impinger. [220,221]. In order to create pMDI formulations, PEG-grafted phospholipids were dispersed, and, subsequently, in situ LPs formed in the impinger’s aqueous environment. [222]. Issues such as complex formulation, poor aerosolized FPF dose, and stability limit LPs formulation delivery via pMDIs [223]. These issues would be more restrictive with the delivery of EVs using pMDIs, which are more biologically complex.

##### Dry Powder Inhalers

Dry powder inhalers (DPIs) are breath actuated, thus avoiding pMDIs problem concerning coordination between inspiration and actuation. A variety of drying techniques, such as spray drying, freeze drying, spray freeze drying, or air jet micronization, have been used to examine the delivery of LPs utilizing DPIs. It has been demonstrated that spray drying of drug-loaded LPs is suitable for generating particles with a small MMAD, i.e., high FPF, as in situ rehydration of LPs occurs following powder deposition in the respiratory tract aqueous environment [224]. Enhancing transfection of gene therapy by introducing a spray-dried lactose solution comprising lipid-polycation-pDNA in comparison with the preparation prior to spray drying [225]. Practically speaking, pro-LPs are more suitable for pulmonary delivery via DPIs, as spray-dried phospholipid formulations can produce LPs immediately once in contact with the aquatic respiratory environment [212]. Compared to LPs, pro-LPs are stable formulations that generate LPs upon hydration with an aqueous phase. Pro-LPs are stable powders of carbohydrate coated or combined with PL, cholesterol, and drug using either small-scale production techniques, e.g., modified rotary evaporators, or large-scale techniques, e.g., fluid-bed coating or spray-drying. [226] Two types of pro-LPs were reported; particulate-based pro-LPs form successful formulation based on the proper selection of an appropriate carrier with porosity and ability to accommodate PLs on its surface [227], and solvent-based pro-LPs successful formulation depends on the use of an appropriate organic solvent that dissolves PLs and at the same time miscible with water [228]. Similarly, in order to expedite the clinical and commercial uses of EVs, freeze-drying as well as spray drying can be employed to preserve their decomposable bio-entities. In the process of freeze-drying, the EVs’ aqueous content is sublimed after it has been frozen, but in the process of spray-drying, the EV-containing solution is first atomized before droplets are quickly transformed into a dry powder using hot gas [229]. Pro-EVs, on the other hand, can be created by atomizing the EV solution in the drying chamber, where the moisture quickly evaporates upon contact with heated air to generate dry particles. The stability of EVs is affected by the atomization pressure and outlet temperature during the process. In comparison to freezing drying, spray drying is a continuous process that can achieve one-step grinding, which is more cost-effective, and extensible, and allows for the adjustment of product particle size [230]. The homogeneity and heterogeneity of EVs give them vast and distinct advantages in disease diagnosis and therapy compared to synthetic carriers like LPs and NPs. However, the therapeutic application of EVs is constrained by their poor targeting, low yield, low purity, and storage stability. Hence, to address the aforementioned issues and permit future applications of EVs, more investigations are required. 

##### Soft Mist Inhalers

Propeller-free metered-dose inhalers called soft mist inhalers (SMIs) produce slow-moving watery aerosols for deep-lung deposition [231]. An example is the Aer_X_^TM^ insulin Diabetes Management System (iDMS^®^; developed by Aradigm [Aradigm Corporation, Hayward, CA]) and Novo Nordisk (Novo Nordisk A/S, Bagsværd, Denmark). It is an inhalation device activated by breathing; it delivers aqueous formulations through micro holes into a slow velocity mist, which permits patient monitoring to guarantee compliance in ideal breathing conditions with a suitable inhalation technique [232]. Since large doses are required to treat lung diseases (e.g., cancers, infectious diseases, etc.) as well as systemic diseases (e.g., diabetes mellitus), all mentioned devices (i.e., pMDIs, DPIs, and SMIs) can only deliver small amounts of aerosol. Thus, they are more suitable for treating diseases (e.g., asthma and COPD) that require therapeutic agents in small doses [233]. The aerosolization of EVs using SMIs would be more valuable than cells. Due to their improved stability when frozen and possibly upon using excipients, stabilizers, and cryoprotectants, EVs are more suited for inhalation treatment.

##### Medical Nebulizers

The most commonly used inhalers for delivering LPs are nebulizers. They generate large volumes of inhalable aerosol, where the formulations employed don’t require drying processes as in DPIs or propellants as in pMDIs [234]. Air jet, ultrasonic, vibrating mesh, and static mesh nebulizers are the four main types of nebulizers. The most widely utilized aerosolized delivery device in the world and the best-recognized nebulizer for LP delivery is the air jet type nebulizer [235]. While the vibrating mesh nebulizer has proven to be exceptionally suitable for delivering vesicles in FPF, the ultrasonic nebulizer has been proven to be the least suitable for the delivery of LPs [236]. While air-jet nebulizers use pressurized gas delivered via a small nozzle to transform medication solution or dispersion into inhalable aerosol droplets, vibrating mesh nebulizers are capable of breaking aggregates into discrete vesicles appropriate for inhalation [237]. In contrast, ultrasonic nebulizers produce more aerosols from a liquid by using ultrasound waves produced by a piezoelectric crystal that vibrates at a high frequency [238]. In order to get around issues with jet nebulizers high production rates and irregular mass distribution, mesh nebulizers were created. They can be divided into passive (static) mesh nebulizers, and active (vibrating) mesh nebulizers, in which the mesh is set on a piezoelectric ring that vibrates [239]. Recent investigations have demonstrated that human adipose-derived mesenchymal stromal cells (MSC-EVs) can be nebulized using vibrating mesh [240], and ultrasonic nebulized hypoxic human umbilical cord MSC-EVs [241] create promising clinical applications in lung injury diseases.

## 6. Applications of Liposomes and Extracellular Vesicles in Pulmonary Drug Delivery

The enormous development of smart NPs has effectively contributed to drug targeting. Ideally, NPs remain non-reactive during their lifetime in circulation and release the drug cargo to the site of action [242]. The most common lipid-based vesicles are LPs that are stably incorporate hydrophilic and/or lipophilic drugs and readily fuse with the cell membrane for intracellular drug delivery. However, LPs still have safety and stability issues, such as mononuclear phagocytic system (MPS) clearance, toxicity at high doses, and host immune reactions upon frequent administration [243]. LPs limitations can be overcome by EVs, which have recently been investigated as a promising drug delivery system [244]. Being naturally occurring lipid-based molecular carriers, EVs show good safety profiles and lower stability issues, as well as do not cause cell death and inflammation upon repeated administration due to their biocompatibility and low immunogenicity [245]. The discovery that EVs have a homing behavior, specifically accumulating within originally producing cells in vivo, opens the way for EVs-based Trojan horse targeting therapies [246].

A hybrid vesicle system with LP and EV advantages (drug binding ability and biocompatibility, respectively), and without or reduced their disadvantages, will effectively and with noticeable target specificity cooperatively deliver a payload to the recipient cells. These hybrids may be prospective customized vectors that can adapt to the pathology of the patient cellular components [247]. EVs are emerging as vital components of multiple chronic respiratory diseases, including asthma, COPD, pulmonary arterial hypertension (PAH), lung cancer, cystic fibrosis, and pulmonary sepsis. Although lung diseases are physiologically complex and heterogenous, EVs and their corresponding cargos have the potential to support the development of innovative lung disease medicines and the identification of clinically meaningful biomarkers. The millions of individuals with lung problems should have some optimism thanks to this exciting area of research that cannot currently be treated with current standards of care [248]. The fact that stem cell-based therapy can restore physiology during pulmonary diseases by acting in a paracrine manner is attributed to the release of EVs that cross-talking with tissues at the location of injury [15].

The difficulty of early diagnosis leads to the development of lung cancer, especially non-small cell lung cancer (n-SCLC), the primary factor in cancer-related fatalities globally. Hence, treatment options, including radiation, surgery, chemotherapy, targeting, immunotherapy, and combination therapy, are ineffective because the late stage of metastases reduces overall survival years [249]. Preclinical and clinical studies offer the potential for the use of EVs in cancer management based on their role in cancer growth and metastasis as biomarkers, in addition to their potential of loading and delivery of antitumor agents as ideal drug delivery platforms [250]. The administration of drug-loaded BioEVs or EVs provides a cell-free therapy that confers advantages concerning production, storage stability, and safety.

Pulmonary delivery of inhalable LPs offers unique advantages as they are comprised of phospholipids comparable to those found in endogenous pulmonary surfactants. The superior therapeutic activity of LPs in the treatment of pulmonary diseases has been proved by laboratory tests and clinical studies [251]. In contrast, EVs generated from different types of lung cells, including epithelial cells, alveolar macrophages, and endothelial cells, are more bio-similar delivery platforms, especially their role in lung diseases that have been studied by their isolation from bronchial-alveolar lavage fluid (BALF) [252,253]. Similar to the particulate-based pro-LPs, engineering hybrid particulate-based pro-EV-LP could expand the potential of pulmonary therapy by targeting intrapulmonary diseases, such as asthma, COPD, PAH, lung cancer, cystic fibrosis, and pulmonary sepsis, to extrapulmonary diseases, such as diabetes mellitus.

In addition, one of the several pulmonary drug delivery devices, DPIs, are the most preferred dosage form due to their better physical and chemical stability and ability to target drug deep into the respiratory tract using the patient’s breathing [254]. Therefore, in order to upgrade the level of inhalation therapy clinical outcomes, a formulation with optimized physicochemical properties is essential [255]. Pulmonary therapy is becoming more patient-compliant due to minimal invasiveness and economic benefits following the scientific and technological advances in DPIs formulations (e.g., Exubera^®^, Technosphere^®^ insulin, AIR^®^ insulin, Afrezza^®^, Inbrija^®^, Adasuve^®^, Levadex^®^, CVT427, possible COVID-19 drugs, and vaccines…etc.) and devices (e.g., Advair Diskus^®^, ProAir^®^ Respiclick^®^, Bero^®^ Ellipta^®^, Tobi^®^ Podhaler^®^, Relenza^®^ Diskhaler^®^, Airduo^®^ Digihaler^®^, Wixela^®^ Inhub^®^…etc.) [256]. Many drug delivery systems (e.g., NPs, MPs, solid lipid NPs, nanostructured lipid carriers, polymer-drug conjugates, macromolecules (dendrimers), lipid vesicles (LPs, pro-LPs), and recently EVs) fulfill a variety of biopharmaceutical requirements, including adequate drug loading, protecting the actives from deterioration, and ensuring biocompatibility, biodegradability, and stability during aerosolization. However, a significant drawback of these systems is their ease of exhalation from the lungs following inhalation [257]. Although LPs for pulmonary delivery have been prevalent for many decades, they are expected to be bypassed by EVs and hybrid vesicles when appropriate formulation techniques are improved. The current part reviews and evaluates the body of literature on respirable LPs and EVs as well as hybrid vesicles.

### 6.1. Inhalable Liposomes

Pulmonary application of LPs is well tolerated owing to LP’s biodegradable lipid backbones, surface charges, lamellarity, and smaller size (nm) that promotes ease of encapsulation into particles with appropriate aerosolization characteristics facilitating optimal deep lung deposition [258]. In addition, the adhesion of LPs to the mucosal surfaces of the airways promotes their accumulation and prolonged retention. Thus, controlled drug release can improve the therapeutic outcome and patient compliance [259] (Table 1). Folic acid conjugated docetaxel dry powder inhalable LPs (DPI-LPs) developed by Zhu et al., 2019, using a thin lipid film hydration method followed by spray-drying with mannitol and leucine showed spherical particles with an average size of 346.80 nm, a zeta potential of −29.30 mV, and encapsulation efficiency (EE) of 99.50%. An in vitro study showed that more than 70% of docetaxel releases slowly within 50 h in phosphate-buffered saline (PBS, pH 7.4). In addition, an in vivo study utilizing rats’ intratracheal administration showed 23.39-fold higher docetaxel deposition within the lung as compared to IV administration at the same dose [260].

DPI-LPs, using trehalose as a lyoprotectant in the lyophilization technique developed by Gandhi et al., 2015, showed an FPF of 56.12%, an emitted dose (ED) of 88.99%, and an MMAD of 3.91 µm, measured at 60 L/min. An in vivo study employing gemcitabine-HCl intratracheal administration showed a marked improvement in area under the curve (AUC) by 8.31-folds and mean residence time (MRT) by 5.69-folds in comparison to the drug alone at the same dose [261]. Oseltamivir phosphate DPI-LPs developed by Tang et al., 2015, using the method of film dispersion, then spray drying showed spherical particles with an average size of 105.90 nm, the zeta potential of −13.65 mV, and EE of 60.43%. An in vitro study demonstrated a sustained pattern for 20 h in PBS, pH 7.4, whereas the drug solution had a release of more than 90% within 2 h. An in vivo study employing intratracheal administration showed a significant improvement in AUC by 1.14-folds and MRT by 1.22-folds in comparison to the drug solution alone at the same dose. This improvement may be due to oseltamivir phosphate prodrug transformation into carboxylate active form within the rat respiratory system by carboxylesterase enzyme [262]. Moxifloxacin DPI-LPs developed by Hamed et al., 2019, using a reversed-phase evaporation method followed by spray drying with dextran, showed corrugated surface and dimple-shaped MPs with an average size of 277 nm, the zeta potential of −12.31 mV, and an EE of 66.25%. An in vitro aerodynamic study utilizing the Aerolizer^®^ device showed a greater respirable proportion, i.e., FPF of more than 75%, whereas in vitro release study revealed a biphasic pattern of release. This is composed of an initial 2 h drug burst release of approximately 50% followed by controlled release up to 48 h of the whole 100% in PBS, pH 7.4. In vivo research employing Penncentury^®^ green fluorescence-labeled microparticles intrapulmonary administration showed sufficient drug deposition within the alveolar macrophages as compared to the upper respiratory tract. This is important because a higher alveolar deposition is important in the treatment of pulmonary tuberculosis (TB) and other disorders [263].

The conventional bronchodilator salbutamol sulfate was developed by Honmane et al., 2019, as DPI-LPs using a thin-film hydration technique followed by spray drying with lactose. The study showed particles with an average size of 167.20 nm, a zeta potential of 9.74 mV, and an EE of 80.68%. During the in vitro dissolution research, a controlled release profile of approximately 90% within 14 h in PBS, pH 7.4, was visible [264]. Ye et al., 2017, designed clarithromycin DPI-LPs using the thin lipid film hydration method followed by ultrasonic spray freeze drying with a combination of mannitol and sucrose as lyoprotectants. At a flow rate of 100 L/min, the aerodynamic investigation revealed an FPF of 43.82% and an ED of 53.78%. The average particle size and EE did not change over the course of a three-month stability investigation at 25 °C and 60% relative humidity [265]. Curcumin DPI-LPs developed by Zhang et al., 2018, using the film hydration method followed by freeze drying, produced irregularly shaped MPs that, during next-generation impactor (NGI) aerodynamic assessment, showed an FPF of 46.71% and an MMAD of 5.81 µm. An in vivo study showed that the curcumin DPI-LPs had more anti-cancer potential following pulmonary administration compared to the drug alone and gemcitabine by regulating tumor enzymatic markers [266]. Khatib et al., 2019, developed DPI-LPs encapsulating ciprofloxacin nanocrystals using a freeze-thaw method followed by spray drying with sucrose as a lyoprotectant. The in vitro aerodynamic study displayed a higher FPF of 69.70%, and controlled release lasting up to 12 h was seen in an in vitro dissolution experiment in (4-(2-hydroxyethyl)-1-piperazineethanesulfonic acid) buffered saline pH 7.4 (HEPES, pH 7.4) [267]. Li et al., 2017, developed andrographolide DPI-LPs using a solvent injection method followed by freeze-drying with mannitol as a lyoprotectant with average particle size of 77.91 nm and zeta potential of −56.13 mV. In vivo study against *S. aureus* intratracheal administration showed adequate antibacterial activity at a dose ten times lower than the drug alone [268].

Inhalable LPs were also formulated to encapsulate phytoconstituents and found to be potential therapies for pulmonary targeting. Viswanathan et al., 2019, fabricated licorice acetone extract loaded DPI-LPs using a thin-film hydration method followed by freeze-drying with trehalose as lyoprotectant and carrier with particles average of 210 nm and an EE of 75%. Utilizing the Lupinhaler^®^ device, an in vitro multistage cascade examination at a flow rate of 60 L/min revealed an FPF of 54.68%, an MMAD of 4.29 m, and a geometric standard deviation of 1.23. More than 46% of the drug was found to be deposited in the lungs of Swiss-albino mice using the nose-only apparatus, compared to 16% that retained in the lungs 24 h after administration. A substantial decrease in bacterial lung count was seen in mice infected with M. tuberculosis as part of an in vivo pharmacodynamic study. Briefly, licorice DPI-LPs were found to be effective against tuberculosis, either on their own or in conjunction with currently available prescribed drugs [269]. Chennakesavulu et al., 2018, used the thin-film hydration approach to create colchicine and budesonide-loaded DPI-LPs, which were then lyophilized using mannitol as a carrier and glycine as an anti-adherent. The results showed that colchicine particles had an average size of less than 100 nm, the zeta potential of −24.7 mV, and an EE of 50.94%, whereas budesonide particles had an average size of less than 100 nm, a zeta potential of −36.9 mV, and an EE of 74.22%. Colchicine and budesonide had an FPF of 44.45% and 48.62%, respectively, at a flow rate of 28.3 L/min, according to an in vitro aerosolization study using the Rotahaler^®^ device. Both particles’-controlled release up to 24 h and adherence to Higuchi’s diffusion-controlled kinetic model in PBS pH 7.4 was demonstrated by an in vitro diffusion study. In addition, in the in vivo study in idiopathic pulmonary fibrosis (IPF) rats, combined particles showed a 1.17 and 3.53-fold reduction in hydroxyproline content, whereas myeloperoxidase activity showed a positive effect against IPF [270]. 

In summary, LPs have been explored as effective inhalable carriers for pulmonary delivery of synthetic drugs, herbal extracts, phytoconstituents, proteins, and peptides for the treatment of lung disorders, including lung cancer, COPD, asthma, and other conditions of the lungs. Also, many conventional and novel techniques for LPs delivery as DPIs have been explored. A proper selection of PL composition and aerosolization technique can generate inhalable LPs capable of retaining their payload and particle size, achieving higher pulmonary deposition and lung retention after inhalation for a prolonged period of time.

**Table 1:** Overview of dry powder inhalable LPs (DPI-LPs) formulations evaluated in in vivo experiments.

Disease	Drug	Study Goals	Liposomes Composition	Drying Instrumentation	Reference
Cancer	Docetaxel-FA (DTX-FA)	Physicochemical, pharmacokinetics and pharmacodynamic properties comparison between LPs-DTx-FA solution and co-spray dried LPs-DTx-FA	-Drug:lipid (1:25 (*w*/*w*)) -PC:Chol (6:1 (*w*/*w*)) -DSPE-PEG-FA: DSPE-PEG-COOH (1:2 (*w*/*w*))	Spray Dryer	[260]
Cancer	Curcumin (CRC)	Physicochemical, pharmacokinetics and pharmacodynamic properties comparison between CRC drug powder, GTB drug powder and freeze-dried LPs- CRC	-SPC:Chol (5:1 (*w*/*w*))	Lyophilizer	[266]
Idiopathic pulmonary fibrosis (IPF)	Colchicine (COL) & Budesonide (BSD)	Physicochemical, pharmacokinetics and pharmacodynamic properties comparison between LPs-COL/BSD solution and freeze-dried LPs-COL/BSD	Drug:lipid (composition) ratio -1:17.5 (*w*/*w*) ≈ 12 mg COL (DPPG:SPC:Chol 3:6:1 (*w*/*w*)) -1:17.5 (*w*/*w*) ≈ 10 mg BSD (DPPG:HSPC:Chol 4:5:1 (*w*/*w*))	Lyophilizer	[270]
Infection	Moxifloxacin (MFX)	Physicochemical, pharmacokinetics and pharmacodynamic properties comparison between mannosylated LPs-MXF solution and co-spray dried mannosylated LPs-MXF	-Drug:lipid (0.15:1 (*w*/*w*)) -PC:Chol (7:3 (*w*/*w*)) -DOTAP:PC:Chol (3.5:3.5:3 (*w*/*w*))	Spray Dryer	[263]
Infection	Clarithromycin (CTM)	Physicochemical, pharmacokinetics and pharmacodynamic properties comparison between LPs-CTM solution and ultrasonic spray freeze dried LPs- CTM	-Drug:SPC:Chol (2:4:1 (*w*/*w*))	Lyophilizer	[265]
Infection	Ciprofloxacin (CFX)	Feasibility of converting LPs-CFX nanocrystals into a co-spray dried LPs-CFX	-SPC:Chol (7:3 (*w*/*w*)) -Sucrose:lipid (2:1 (*w*/*w*))	Spray Dryer	[267]
Infection	Andrographolide (AGL)	Physicochemical, pharmacokinetics and pharmacodynamic properties comparison between AGL drug powder and freeze-dried LPs- AGL	-SPC:Chol (6:1 (*w*/*w*))	Lyophilizer	[268]
Infection	Licorice extract (LR-E)	Physicochemical, pharmacokinetics and pharmacodynamic properties comparison between LPs-LR-E solution and freeze-dried LPs-LR-E	-Drug:lipid (1:6, 7, 8, & 9 (*w*/*w*)) -Lipid:trehalose (1:4 (*w*/*w*))	Lyophilizer	[269]
Influenza	Oseltamivir phosphate (OTV-P)	Physicochemical, pharmacokinetics and pharmacodynamic properties comparison between LPs-OTV-P solution and co-spray dried LPs-OTV-P, OTV-carboxylate plasma concentration	-Drug:LPs (1:10 (*w*/*w*)) -Ovelecithin:Chol (6.7:1 (*w*/*w*))	Spray Dryer	[262]

FA: Folic acid, LPs: Liposomes, PC: Phosphatidyl-Choline, chol: cholesterol, DSPE: 1,2-Di-Stearoyl-Phosphatidyl-Ethanolamine, PEG: Polyethylene Glycol, DOTAP: 1,2-Di-Oleoyl-3-Trimethyl-Ammonium-Propane, SPC: Soybean Phosphatidyl-Choline, DPPG: 1,2-Di-Palmitoyl-sn-glycero-3-Phospho-Glycerol Sodium, HSPC: Hydrogenated Soya-Phosphotidyl-Choline.

### 6.2. Inhalable Extracellular Vesicles

Pulmonary application of EVs showed promising results by studying BALF-derived EVs and their role in diverse lung diseases. BALF is an ideal “lung liquid biopsy” that contains EVs, as it is fluid obtained by flexible bronchoscopy during bronchoalveolar lavage. The EVs that are present in the BALF is secreted from several cells of the airways, including bronchial epithelial cells, endothelial cells, alveolar macrophages, and other immune cells. BALF contains a lot of various EVs, estimated by nanoparticle tracking analysis (NTA) as 1.8–3.8 × 10^8^ compared to 4.0–9.8 × 10^8^ particles per ml in plasma. Interestingly, fluorescence labeling against EV tetraspanins demonstrates that most of the particles found in BALF are “real” EVs, while most of the particles in plasma are non-EV components like lipoproteins. Although EVs are often obtained from plasma and serum, since they are easier to access, BALF appears to be a better source owing to its close closeness to the lung microenvironment in the case of lung disorders [253]. In contrast, the synthesis of LPs from endogenous PLs of the lungs would be biocompatible with the potential to load a wide range of drugs. However, these LPs have a quick metabolism and a fast rate of turnover due to the continual removal and replacement of pulmonary surfactant, with turnover time from type II pneumocytes of the alveoli predicted to last for about 10 h [271]. In addition, pulmonary endothelial cell-derived EVs are essential for maintaining lung homeostasis [272] and are frequently disrupted in a variety of lung disorders [273,274]. As a novel drug delivery vehicle, EVs are natural lipid vesicles capable of protecting, stabilizing, and properly transporting active cargo to the recipient [158,275,276]. The superiority of EVs over LPs, especially in lung targeting, is attributed to their proven stability, reduced offensiveness, and increased uptake into target cells [277] (Table 2). Wu et al., 2016, studied the anticancer activity of EVs derived from curcumin-pretreated lung cancer cells, where transcription factor 21 (TCF21) overexpression inhibits the growth of tumors in a mouse tumor model H1299 [278]. Guo et al., 2019, studied the cell membrane-derived microparticles (CMPs), a type of 100–1000 nm EVs formed directly by cellular membrane shedding in response to diverse physiological and artificial stimuli. Similar to EVs, CMPs also act as intercellular messengers and based on their origins and properties, have innate tissue tropism that can be exploited to target disease organs. CMPs are desirable DDS due to their theoretical biocompatibility, selective tropism, decreased clearance, enhanced penetration of biological barriers, and concomitant increased drug transport to target tissues [279]. Using source cell membrane surface antigens to leverage the innate homotypic adhesion capabilities of CMPs emerging from cancer cells, which can be employed for drug delivery applications, enables cancer cell-specific targeting. More than 85% of lung tumors are n-SCLC, and 40% of this percentage ends with a malignant pleural effusion (MPE) caused by pleural metastasis. To address concerns relating to immunological compatibility and tumor heterogeneity, autologous malignant cells in MPE have been chosen as donor cells for the creation of autologous tumor CMPs (ATCMPs) loaded with methotrexate (ATCMPs-MTX). In patients with advanced lung cancer, the potential of ATCMPs-MTX dual functional technology as a chemo-immunotherapeutic drug via intrapleural delivery lowered MPE volume with clinical benefits and negligible methotrexate-induced toxicity [279]. Kim et al., 2016, harvested EVs from RAW264.7 autologous macrophage condition medium and loaded them with paclitaxel (PTX) by sonicating PTX-EVs mixture. Intranasal administration of PTX-EVs to Lewis lung cancer mice model showed almost complete localization within lung cancer cells and metastases growth inhibition compared to the drug alone [164]. Furthermore, these PTX-EVs were modified by incorporating aminoethylanisamide PEG to boost their capacity for drug loading and hence therapeutic activity [161].

Asthma is a chronic inflammatory condition characterized by hyperresponsive airways to triggers such as viruses, allergens, and exercise that exaggerate airway-narrowing. Mesenchymal stem and regulatory T cells (Tregs) are powerful immunomodulators. Yumo et al., 2018, investigated how MSCs-EVs promoted Tregs proliferation and immunosuppression by upregulating IL-10 and TGF-1 from peripheral blood mononuclear cells (PBMCs) of asthmatic patients. This discovery clarifies MSCs-EVs’ potential for treating asthma [280]. Similar findings were made by Cruz et al., 2015, who discovered that human bone marrow-derived MSCs-EVs are equally or more effective than murine bone marrow-derived MSCs-EVs in reducing both Th2-mediated eosinophilic and Th2/Th17 neutrophilic-mediated allergic airway inflammation in mice induced by repeated airway mucosal exposure to Aspergillus fumigatus hyphal extract [281].

IPF is a prototype of chronic, progressive, and fibrotic lung disease of unknown etiology with an average survival period of 3–5 years. Destroyed alveolar architecture and altered extracellular matrix replace the healthy tissue, which causes disruptions in gas exchange, decreased lung compliance, and ultimately respiratory failure and death [282]. Pirfenidone and nintedanib, two antifibrotic drugs, can only delay the progression of the disease with significant side effects, and in many cases, the future of the treatment is unknown [283]. Shentu et al., 2017, studied in vitro the effect of IPF patient’s lung fibroblasts uptake of BM-MSCs-EVs in downregulating TGF-β1, a pathogenic factor that induced myofibroblastic differentiation [284]. Dinh et al., 2020, showed that inhalation of human lung spheroid cells’ (hLSCs) EVs is superior to their MSCs counterparts in reducing fibrosis, apoptosis, and collagen deposition and restoring pulmonary function in a rat model of bleomycin-induced pulmonary fibrosis [285]. In a similar manner, Royce et al., 2019, discovered that co-administration of the antifibrotic medication serelaxin improved the therapeutic efficacy of human amnion epithelial cells (hAECs) EVs in treating basement membrane-induced fibrosis and associated airway dysfunction [286].

The increasingly prevalent condition COPD, which is brought on by smoking, is expected to overtake all other causes of mortality in the next ten years. Small airway inflammation and increasing parenchymal damage are the main symptoms of the illness, which also cause lung tissue loss and obstructive pulmonary dysfunction because of gas entrapment, inadequate expiratory flow, and reduced gas exchange [287]. Currently, corticosteroid immunosuppression is the standard treatment for symptomatic relief of COPD. Although further clinical investigations are required, the administration of MSCs-EVs seems to be a safe and feasible option [288]. EVs provide better therapeutic results than traditional medicines, suggesting they could be a unique therapeutic option for CRDs. This is evidenced by in vitro studies utilizing rodent models, where MSCs-EVs alone or in combination with therapeutic drugs have improved the therapeutic potential of patients’ CRDs.

**Table 2:** Overview of EVs formulations evaluated in in vivo experiments.

Disease	Drug Loading/Bioengineering	Study Goals	EVs Extraction	Reference
Cancer	A 10 mM solution of CRC powder dissolved in DMSO and added to the growth medium for intervals of 24 to 72 h.	-According to BCS, CRC belongs to a category IV drug (i.e., low solubility—low permeability) as well as CRC poor stability and rapid elimination paving the way for novel delivery systems [289]. Then, due to higher CRC concentrations in recipient cells, CRC, which is released by EVs, exerts a stronger anti-cancer effect. -Pharmacokinetics and pharmacodynamics comparison between CRC drug alone and EVs-CRC	Differential centrifugation	[278]
Cancer	After treating with MTX, tumor cells were exposed to UV light, 300 Jm-2 irradiations for various period of times for different cell types. After 24 h of incubation, supernatants were taken out and repeatedly centrifuged to remove cells, debris, and lastly to pellet ATCMPs.	-In vitro cells cytotoxicity assay and MPE mice model intrapleural injected with PBS, MTX drug alone, naïve empty ATCMPs, or ATCMPs-MTX. In contrast, 11 human MPE patients’ autologous tumor cells obtained via indwelling pleural catheter to produce ATCMPs to package MTX for individualized MPE therapy. -In vitro and in vivo comparing the pharmacokinetics and pharmacodynamics of the MTX alone and ATCMPs-MTX malignancy targeting.	Differential Centrifugation	[279]
Cancer	Loading Paclitaxel (PTX) into EVs released by autologous macrophages were followed three methods; incubation, electroporation, and sonication.	-Physicochemical comparison among different drug loading into EVs methods; incubation, electroporation, and sonication. The highest loading efficiency was achieved with mild EVs sonication in the presence of PTX. -In vitro and in vivo (intranasally (i.n.) administered in murine model of tumor lung metastases) pharmacokinetics and pharmacodynamics cytotoxicity comparison between PTX drug alone and EVs-PTX.	Polymer Precipitation (ExoQuick-TC™ Kit)	[164]
Cancer	First: PTX was added to EVs in PBS. Second: different amounts of aminoethylanisamide-polyethylene glycol-DSPE (AA-PEG-DSPE) were mixed with the EVs-PTX combination. Third: the final mixture was sonicated to obtain a solution of AA-vectorized EVs loaded with PTX (AA-PEG-EVs-PTX).	-Development and optimization of a formulation of AA-vectorized EVs superior structure loaded with PTX (AA-PEG-EVs-PTX) target the sigma receptor, which lung cancer cells overexpress. -In vitro and in vivo (intravenously (i.v.) administered in murine model of tumor lung metastases) pharmacokinetics and pharmacodynamics comparison between autologous vectorized labeled (DiL-AA-PEG-EVs-PTX) and non-vectorized labeled (Dil-PEG-EVs-PTX) concerning prolongation circulation time via PEGylation, targeting/accumulation via AA-vectorization, and bypassing Pgp (P-glycoprotein efflux pump)-mediated drug efflux in MDR cancer cells.	Polymer Precipitation (ExoQuick-TC™ Kit)	[161]
Idiopathic pulmonary fibrosis (IPF)	Human bone-marrow derived mesenchymal stem cells EVs (hBM-MSCs-EVs).	-Proving that normal and IPF lung fibroblasts’ TFG-1-induced myofibroblastic differentiation is suppressed by hBM-MSCs-EVs and not by fibroblast EVs. -Evaluating cellular EVs uptaking kinetics, hBM-MSCs-EVs exhibit higher time- and dose-dependent cellular uptake compared to fibroblast EVs. Contrarily, Thy-1 removing or blocking as well as Thy-1-beta integrin interactions inhibiting reduced the hBM-MSCs-EVs uptake and thereby avoided suppressing of myofibroblastic differentiation.	Differential Centrifugation	[284]
Idiopathic pulmonary fibrosis (IPF)	Human lung spheroid cells EVs (hLSCs-EVs) and Human bone-marrow derived mesenchymal stem cells EVs (hBM-MSCs-EVs).	-A viable way to mitigate cell-based therapy clinical challenges, is to substitute conditioned medium or secretome for real cells. Thus, stem cell’s regenerative ability via paracrine activity can be gained through their secretions, i.e., secretome and EVs. -Demonstrating that mice model of BLM/silica-induced fibrosis, nebulizer inhalation of hLSCs-EVs promotes lung repair superior to hBM-MSCs-EVs.	Differential Centrifugation	[285]
Idiopathic pulmonary fibrosis (IPF)	Human amnion epithelial cells derived EVs (hAECs-EVs)	-Although stem cell-derived EVs offer several therapeutic advantages over their parenteral cells, their therapeutic effects can be impaired by fibrosis. -In vivo treatment efficacy comparison on OVA/NA induced chronic AAD and BLM induced pulmonary fibrosis mice model between hAECs-EVs alone and hAECs-EVs + serelaxin (SLX).	Differential Centrifugation	[286]
Asthma	Human bone-marrow derived mesenchymal stem cells EVs (hBM-MSCs-EVs).	-Examining the role of hBM-MSCs-EVs paracrine effects in immune modulation that mimics paternal MSCs and hence therapeutic potential for asthma. -hBM-MSCs-EVs promote Tregs propagation and immunological suppression capacity by upregulating PBMCs cytokines IL-10 and TFG-β1 of asthmatic patient.	Differential Centrifugation	[280]

BCS: Biopharmaceutical Classification system, MTX: Methotrexate, ATCMPs: Autologous Tumor Cell Membrane-derived MicroParticles, MPE: Malignant Pleural Effusion, PBS: Phosphate Buffer Saline, DSPE: 1,2-Distearoylphosphatidylethanolamine, Dil: Tetramethylindocarbocyanine Perchlorate, Tregs: Regulatory T cells, PBMCs: Peripheral Blood Mononuclear Cells, Thy-1: Thymus cell antigen 1, BLM: Bleomycin, OVA/NA: Ovalbumin/Naphthalene, AAD: Allergic Airway Disease.

### 6.3. Inhalable Hybrid Vesicles

Technological advances in nanomedicine concerning improved DDSs development during recent years have all been made possible via adding targeting ligands, proteins, or PEGylation. However, a large variety of synthetic NPs still have concerning limitations due to their low stability, inability to bypass the immune system and overcome biological barriers, dose-limiting toxicity, immunogenicity, and limited targeting profile [290,291]. Recently, EVs-based DDSs have become very popular due to their possible inherent advantages, such as proposed preferential accumulation to particular cells and organs, low immunogenicity [292], and proposed ability to cross biological barriers [293,294], properties that are not well found in synthetic DDSs. Thus, naturally, protein-rich EVs membrane is of paramount importance to be considered as novel bioinspired nanoplatforms that can outperform synthetic DDSs. However, there are still several difficulties in developing EVs as DDSs, chiefly the absence of standardized techniques for effectively loading them with medicinal cargo [295,296]. 

The combination of natural EVs and synthetic NPs, such as LPs using well-defined techniques leads to the formation of hybrid vesicles as novel nanoplatforms for drug delivery. Choosing an appropriate strategy depends on many considerations, such as reproducibility, size of hybrid vesicles, stability, minimization of byproducts, and preservation of carrier function. Strategies employed to serve this goal include passive hybridization [297,298,299], the transient opening of lipid bilayers [300,301,302,303,304,305], and the fusion of lipid bilayers [50].

Hybrid vesicles will improve the merits of both natural and synthetic NPs. These merits include immune evasion [306,307,308,309], overcoming biological barriers [310,311,312,313,314], enhanced cellular uptake and cargo delivery [315,316,317,318,319], and homing properties [320,321,322,323]. In one study, novel hybrid EVs-LPs systems were designed and produced by the fusion of fibroblast-derived EVs with LPs containing clodronate (EVs-LPs-CLD) or drug-free (EVs-LPs). Both hybrids showed a greater ability to penetrate lung fibrous tissue in mice, 3.4- and 1.8-fold, respectively, compared to LPs [305]. Since the cell source appears to have an impact on the in vivo biodistribution of EVs [324], it is possible that the presence of certain proteins on the membrane of EVs is what causes the improved interstitial penetration of hybrid EVs-LPs. Therefore, using EVs’ inherent capacity to traverse biological barriers may help ensure that therapeutics are delivered precisely to physiologically protected locations after hybridization.

## 7. Models for Testing Inhaled Aerosolized Mist/Dried Lipid Bilayer Vesicles 

**Figure 12:**
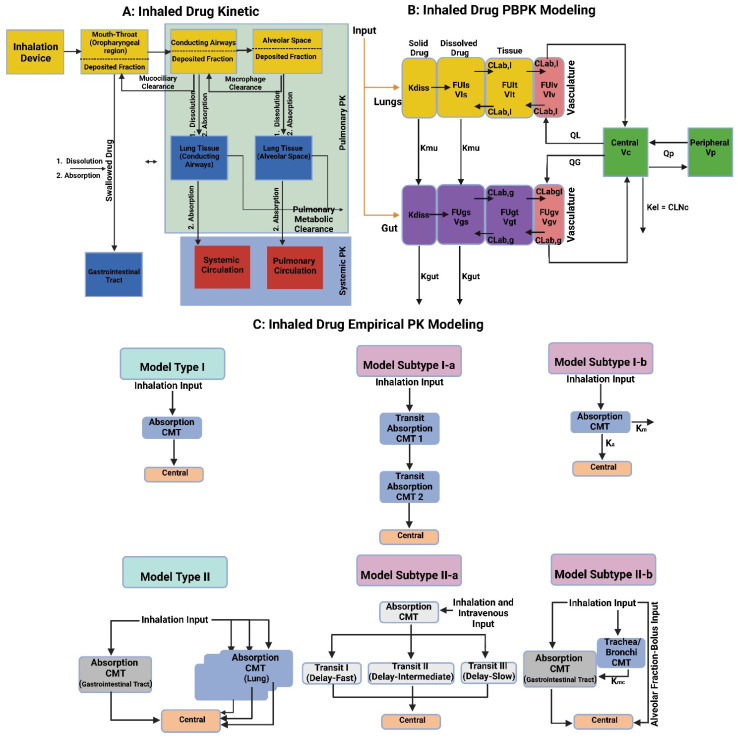
Schematic representation: (**A**) Inhaled Drug Kinetic. (**B**) Inhaled Drug PBPK Modeling. (**C**) Inhaled Drug Empirical PK Modeling.

The lung is a complex organ with structures and types of cells dedicated to accomplishing its particular physiological functions. Nevertheless, research and development of inhaled drugs for the efficient local and systemic management of many diseases are still ongoing. In recent years, the importance of mathematical modeling to evaluate and comprehend pharmacokinetics (PK) in clinical drug development has increased. PK models can be categorized into physiologically based (PBPK; PK parameters derived from the physicochemical and physiological inhaled drug characteristics, called bottom-up approach) and empirically based (PK parameters estimated based on clinical data, called up-bottom approach) models that can be explored mechanistically. Pulmonary PK refers to lung-specific kinetics prior to drug absorption into the systemic circulation, such as pulmonary dissolution, pulmonary absorption, or mucociliary clearance, whereas systemic PK refers to PK processes following drug absorption from the lung into the systemic circulation [325] (Figure 12).

For both local and systemic diseases applications, successful inhaled drugs discovery and development increasingly demand an essential preclinical task to assess and predict pulmonary deposition and absorption, e.g., local drug retention, systemic absorption, and in vitro-in vivo correlation/extrapolation (IVIVC/E) [326]. Thus, preclinical methods for testing lung absorption in vitro, ex vivo, and in vivo continue to advance thanks to several technical, methodological, and analytical improvements. In vitro lung epithelial cell monolayer-based models have been used to evaluate transepithelial drug transport only, while their kinetic relevance to in vivo lung absorption has yet to be demonstrated. The ex vivo isolated perfused rat lung (IPRL) paradigm allowed for more precise kinetic and mechanistic elucidation of lung tissue/organ-level deposition and absorption since it was a compromise between in vivo and in vitro models. However, with only a 3 h viability, it was not suitable for assessing delayed lung absorption of big macromolecules. In contrast, in vivo studies using small rodents have been modified in order to characterize systemic PK profiling with improved lung dose methodologies and bioanalytical methods [327]. This section highlights the variability between in vitro, ex vivo, and in vivo models with respect to inhaled drugs pulmonary and systemic PK modeling.

### 7.1. In Vitro Pulmonary Cell-Based Models

The 3R plan, which stands for (Replacement, Reduction, and Refinement) urges a decrease in the use of animals, the creation of non-animal protocols, and the advancement of scientific approaches to animal welfare. This has led to the acceptance of cells as alternatives to animal models and due to other factors, such as cost reduction, predictability and simulation, and rapid throughput [328]. In addition, cells mimic the tissue microenvironment, and hence lung cells have been widely employed to study inhaled drug absorption, transport, and metabolism [329]. For inhaled biopharmaceutics research, lung epithelial cells that represent the rate-limiting transport barrier for inhaled pharmaceuticals are reconstructed under cell culture in the Transwell/Snapwell system as confluent polarized monolayers lung epithelial cells. The rate (not the extent) of test molecule transepithelial diffusion across the lung cell monolayers is represented by the apparent permeation flux (J_app_) or the apparent permeability coefficient (P_app_). The lower values can be predicted upon local delivery, i.e., longer lung retention/residence, whereas the higher values favorably indicate systemic delivery [330,331]. 

**Figure 13:**
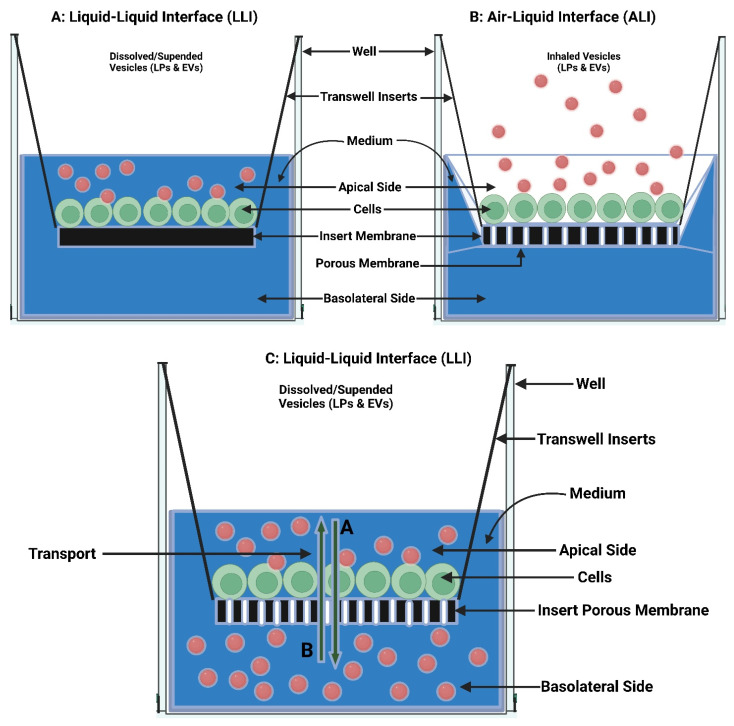
Schematic representation of cell exposure models employed for studying cells-vesicles interaction: (**A**) Liquid-liquid interface (LLI). (**B**) Air-liquid interface (ALI). (**C**) Liquid-liquid interface (LLI) includes a porous membrane.

For studying the pharmacokinetics-pharmacodynamics (PK-PD) of inhaled aerosolized mist/dried lipid bilayer vesicles (LPs and EVs), the pulmonary cells are grown under submerged liquid-liquid interface (LLI) or air-liquid interface (ALI) conditions. Although ALI exposures are more physiologically realistic and so potentially more biologically relevant than submerged LLI exposures, the latter are experimentally simpler. In the LLI condition, the interaction between submerged cells and inhaled vesicles takes place after the vesicles dissolve or are suspended in the cell culture media. In contrast, in the more realistic in vivo ALI condition, the interaction between cells and inhaled vesicles occurs after the vesicles deposition onto the cultured cells exposed to the inhaled air from one side while being in contact with the hypothetical blood circulation of the cell culture from the other side. Despite the experimental simplicity, submerged LLI cell exposures have two major limitations. First, the dose of cells-vesicles interaction is often unknown because the vesicles fraction that reaches cells can’t always be detected or calculated from the vesicle’s hydrodynamic properties (size, density, shape) primarily for vesicles smaller than 100 nm (LPs and EVs average size is ≈100 nm) as the diffusion takes over as the primary transport mode. Second, submerged LLI cell culture conditions are created in an unrealistic and artificial environment for alveolar cells in the lungs [332]. To study molecules transportation across alveolar epithelial cell monolayer as well as metabolism, cells are cultivated in Transwell with permeable filter support [327] (Figure 13).

**Figure 14:**
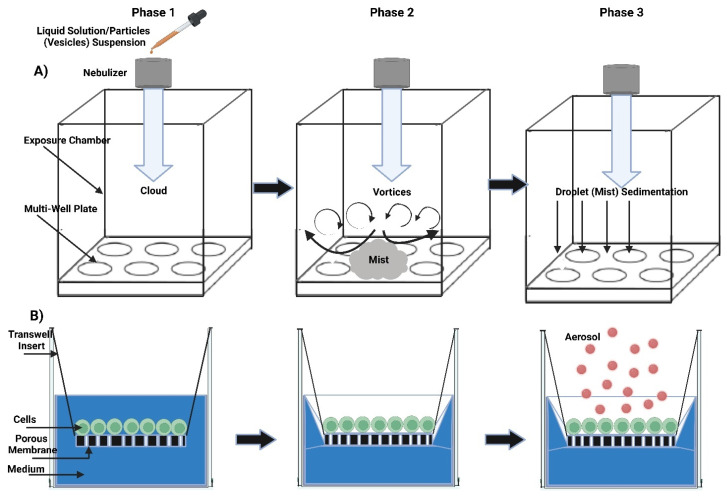
Schematic representation: (**A**) Nebulization and sedimentation of liquid solution/particles (vesicles) suspension using ALICE-CLOUD technology. (**B**) Cell cultivation on transwell inserts.

A novel dose-controlled ALI cell exposure (ALICE) system has been established for studying the effects of NP aerosols (LPs and EVs). In comparison to the submerged LLI and ALI, ALICE consists of four primary parts: a droplet generator (nebulizer), an exposure chamber, a flow system with an incubation chamber that provides temperature and humidity conditions suited for cell development, and a quartz crystal microbalance for real-time measurement of the cell-delivered NP dose. A dense cloud of aerosols is generated by nebulizing a 1 mL suspension with a vibrating mesh nebulizer into the exposure chamber, where it gently deposits due to single particle sedimentation and cloud setting (i.e., aerosols cloud moves as a bulk object rather than a collection of individual aerosols) onto cells cultured at the ALI in cell culture plates [333]. Schmid et al., 2017 studied aerosolized ciclosporin-A LPs in vitro biokinetic temporal profile in the apical, basal, and cell compartment using a dose-controlled ALICE system of human lung epithelial barrier up to 24 h. The biokinetic evaluation showed that lung epithelial cells formed a narrow but imperfect septum resulting in initially trans-barrier ciclosporin-A LPs transport rates that ceased after 4 h. Although this intrinsic transport was observed, a 150-fold higher concentration of ciclosporin-A LPs was generated in the apical cell compared to the basal compartment. For pulmonary targeting, a high cellular ciclosporin-A LPs delivered dose of about 25% was obtained rapidly within less than 1 h and maintained for at least 24 h. This shows that the ALICE system, when used in conjunction with ALI-cultivated lung epithelial cells, offers a dependable and pertinent in vitro platform technology to investigate the effects of inhaled vesicles in PDD under bio-imitated circumstances [334]. ALICE-CLOUD technology is a reliable tool for screening aerosolized drugs due to the principle of cloud motion for quick and efficient delivery of aerosolized liquid drugs onto cultured lung cells under realistic ALI circumstances. This is because a vibrating mesh nebulizer generates very dense cloud of droplets, which behaves as a high-density fluid moving in a low-density fluid [335,336,337] (Figure 14) (Table 3).

The standard exposure condition of LLI represents cell cultivation submerged in the culture medium with no true exposure to the air. In contrast, the ALI exposure condition represents relevant pulmonary cell culture modeling to humans in reality. The ALI has wide applications in cell culture models, e.g., skin cells, corneal cells, gingival epithelial mucosa, and various types of respiratory tract cells, as well as combinations of cell types, e.g., skin with melanocytes, immune cells, and lung cells (triple- and tetraculture models comprising endothelial and epithelial cells, macrophages, dendritic cells, and mast cells). Cell sources have included humans, chickens, and rodents. Applications have covered a variety of topics, including the effects of MPs/NPs, viruses, and bacteria, as well as other chemicals, pharmaceuticals, and tobacco products. Thus, in any form, the ALI resembles in vivo exposure circumstances in a way that is significantly more applicable than current cell culture methods. However, in order to be relevant and practical, aerosols testing needs more extensive experimental techniques, including new engineered-physical advances [338]. Currently, ALI models are more relevant in vivo and appear undefeatable for testing the inhalable effects of airborne substances than any available in vitro submerged cell cultures approach.

### 7.2. Ex Vivo Lung Tissue/Organ-Based Models

Byron et al. pioneered the design of the isolated perfused rat lung (IPRL) preparation that has become the ex vivo lung tissue/organ model employed in inhaled biopharmaceutics research for systemic and local delivery [339,340,341]. After isolation from rodents (rats or mice), the lungs were suspended vertically in a custom ex vivo controlled artificial water-jacketed glass thorax maintained at 37 °C. A physiological buffer solution instead of blood was used to sustain the vascular circulation by using peristaltic pumps in a recirculation or single-pass mode [342]. In other words, lung breathing guarantees physiological relevance, while the IPRL model’s complete vasculature enables examination of drug fate without the interference of extra-pulmonary processes like hepatic clearance. An exteriorized tracheal cannula was used to give drugs to the IPRL as an aerosol, spray, or solution. The proportion of drug mass absorbed profiles from the lung was calculated using the perfusate samples over time, and the apparent absorption rate constants or half-lives were then derived. The higher absorption rate constant and shorter half-life is indicative of systemic delivery, whereas transforming these profiles via mass balance to those of lung retention can be used to reason local pharmacologic actions. However, most studies pertain to low molecular weight drugs because their viability is limited to only 3 h, and this does not support the evaluation of slow lung absorption of macromolecules [343]. The IPRL is an ex vivo tool that preserves lung architecture and function for determining the rate and extent of compounds that are absorbed by the lungs after intratracheal instillation without interfering with whole-body issues like distribution, metabolism, and elimination. IPRL has been used widely to assess rates of lung retention and absorption between substances. Thus, unlike in vitro pulmonary cell-based 2D/3D models, ex vivo lung tissue/organ-based 3D models should allow the in vivo-relevant lung tissue/organ-level kinetic assessment of absorption and deposition.

Current studies demonstrated the ex vivo-in vivo correlations of PDD kinetics, such as absorption, elimination, and pre-epithelial events, including rate-limiting dissolution and release in the lung, as well as extrapolation and prediction between ex vivo and in vivo. Selg et al., 2013, compared lung-specific PK of inhaled dry powder corticosteroid fluticasone furoate (FF) in the IPRL and the endotracheally incubated rate (EIR). The DustGun aerosol generator produced FF aerosols with MMAD ranging from 2.2 to 3.2 µm. The perfusate from the IPRL’s single-pass inhalation of 5.6 and 46 µg of FF was repeatedly measured for 100 min. In contrast, four venous blood samples were taken from EIR who had been exposed to 7 µg of FF by inhalation for up to 4 h. Both IPRL and EIR show FF slow declining, a pulmonary retention half-life of 4.3–4.9 h, and maximum plasma concentration (Cp_max_) of 1.0 and 0.8 nM for the IPRL and EIR, respectively. These results indicate that PK ex vivo-in vivo joint studies can give an in-depth description of inhaled drugs [344].

Ong et al., 2014, employed in vitro and ex vivo complementary methodology for ciprofloxacin (CFX) in vivo pharmacokinetics prediction concerning drug pulmonary disposition and retention. Three CFX formulations were tested; one is LPs-CFX which was consistent across in vitro and ex vivo techniques and predicted in vivo profiles [345]. By comparing a large number of both existing and newly designed substances, Edwards et al., 2016, developed and tested a novel Quantitative Structure-Activity Relationship (QSAR) in an in silico model to improve the physicochemical drivers for understanding pulmonary absorption, thereby facilitating compound design by improving absorption prediction. The QSAR model was built based on pulmonary absorption data using IPRL, as it performed very well in the “Test set” with an observed vs. anticipated correlation of R^2^ = 0.85 and >65% of compounds correctly categorized. As a result, pulmonary absorption is favorably connected with the computed descriptors related to permeability and hydrophobicity, while those related to charge, ionization, and size are negatively correlated. For rating and categorizing compounds before synthesis, this innovative QSAR model can replace the conventional generation of IPRL model data [346].

**Figure 15:**
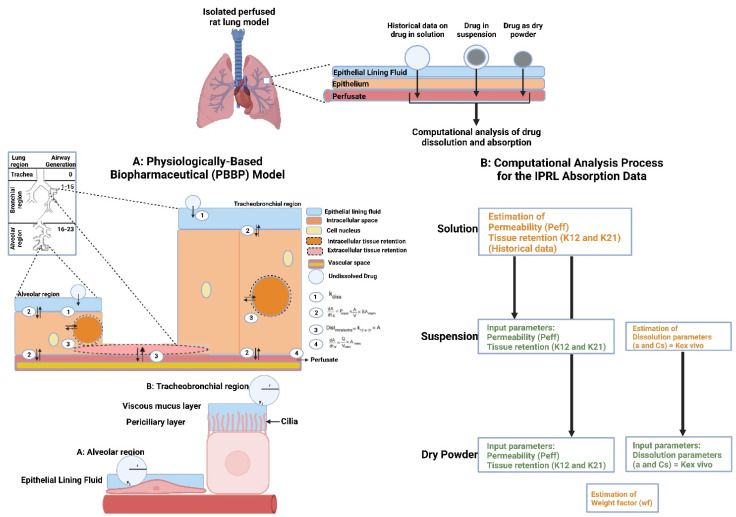
Schematic representation: (**A**) Physiologically-based biopharmaceutical (PBBP) model for (K_diss_) estimation. (**B**) Computational analysis for IPRL (K_absorp_) estimation.

Similarly, Eriksson et al., 2019, developed an in silico physiologically based biopharmaceutical model to assess the experimental IPRL dissolution data (K_ex vivo_) and compared these data with an analogous in silico approach applied to in vitro dissolution data (K_in vitro_). Based on the fact that many inhaled drugs are not very water soluble, and the rate-limiting step of the overall absorption process is the dissolution, the objective of the study was to increase knowledge of pulmonary drug dissolution. This was performed by comparing the pulmonary absorption rates of poorly soluble inhaled drugs from suspensions and dry powders with historical absorption data for solutions to understand the effects of dissolution on the overall pulmonary absorption process. Administering drugs as suspensions, dissolution is the rate-limiting step in the total pulmonary absorption process due to the larger particle size and, consequently, the smaller surface area. There was good agreement between K_ex vivo_ and K_in vitro_, as the projected dissolution parameters were graded in accordance with the solubility of the drugs. On the other hand, the dry powders of all drugs were absorbed more slowly than their suspensions, indicating that wetness is a critical factor in the ability of the dry powders to dissolve. Therefore, a wetting agent was added to the in silico model to explain the variance in absorption patterns between the suspensions and dry powders. Combining IPRL and in silico models is considered a helpful method for learning more about pulmonary drug dissolution and dissolution-related factors for poorly soluble inhaled drugs [347,348] (Figure 15) (Table 4).

Eriksson et al., 2020, investigate the potential of IPRL data (ex vivo input factors) combined with IV-determined PK data biopharmaceutics model to predict experimental rat in vivo plasma concentration-time profiles and lung quantity after inhaling various inhaled drugs. The performance of simulations using ex vivo input parameters was compared to simulations using in vitro input parameters to establish whether and to what degree predictability may be enhanced using input parameters determined from the more sophisticated ex vivo model. Simulations utilizing ex vivo input parameters outperformed significantly better than simulations using in vitro input parameters in predicting in vivo lung absorption [349]. The obtained IPRL data show better absorption parameters for predicting in vivo lung absorption of both solution and suspension formulations than those established from conventional in vitro experiments. Thus, it would be beneficial to base predictions of inhaled drug performance on IPRL data rather than on in vitro data during the drug development process to improve the mechanistic understanding of pulmonary drug absorption processes and to gain a better understanding of how various drug properties and formulations may affect in vivo behavior of inhaled compounds.

### 7.3. In Vivo Whole Animal-Based Models

**Figure 16:**
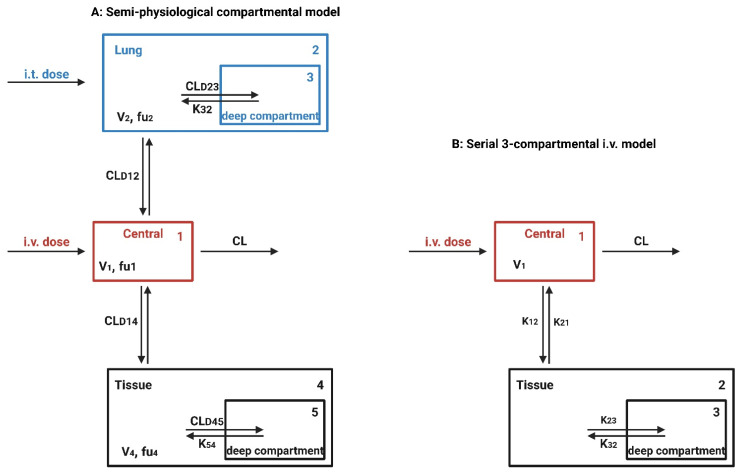
Schematic representation: (**A**) Structure of the compartmental model. (**B**) Serial three-compartmental model.

Compared with microsyringe instillation of drugs to the surgically-exposed trachea, recently, non-surgical pulmonary administration as aerosol-like via orotracheal access has been used to examine the drug’s systemic PK profiles from blood samples. Drugs were spray-instilled orotracheally as 10–100 μL of solution or suspension or insufflated as powders in a typical size of 16–22 μm using specialized or commercial equipment such as PennCentury’s MicroSprayer and Dry Powder Insufflator, and with the use of a laryngoscope [350]. Accordingly, these small rodent studies provided PK profiles for a number of drugs, explaining their human dosages and PK findings for both systemic and local delivery. Recently, attempts have been made to predict the human PK profiles and systemic exposures to inhaled drugs from in vivo rodent PK data following pulmonary delivery and a kinetic modeling approach with allometric scaling. This is based on the persumption that lung absorption in humans and small rodents like rats behave in the same way kinetically. Jones and Harrison 2012 employed kinetic modeling with allometric scaling to estimate human PK profiles after inhaled powder delivery using the apparent drug’s pulmonary absorption rate constants in rats following intratracheal powder insufflation. The predicted Cp_max_ values did not match well with the anticipated Cp_max_ values, necessitating more systematic improvement to the prediction [351]. Hendrickx et al., 2018, argued that allometric scaling should be used in translational compartmental modeling to predict human PK profiles from cross-species scaling of rat PK profiles after intratracheal solution delivery. The effects of the inhaled drugs are surprisingly comparable between the observed and projected human Cp_max_ values [352] (Figure 16) (Table 5).

Although this field of study is very young, it should be quite intriguing. Currently, the ex vivo IPRL model can be employed to determine the in vivo kinetic properties of pulmonary absorption in small rodents by estimating their correlation with P_app_ values of the in vitro cell-based models based on respective drugs’ physicochemical properties with well-predicted in vivo-to-human induction approach. The development of inhaled drug products for local and systemic delivery is aided by methodological, technical, and analytical advancements in preclinical in vitro, ex vivo, and in vivo models of pulmonary absorption. The primary ALI-cultured 3D human lung cell barriers are now available, along with the efforts to incorporate aerosol drug deposition into the in vitro lung cell models are ongoing, where “lung-on-a-chip” technology and stem cell-derived lung epithelial cells are emerging. Due to their capacity for kinetic determination of diffusive tissue/organ-level, membrane protein-mediated absorption and competing non-absorptive loss; evaluation of ‘pre-epithelial’ aerosol biopharmaceutical events in the lung, such as dissolution and release; and extrapolation of ex vivo-to-in vivo data and prediction of pulmonary absorption and disposition, ex vivo IPRL methods have become more and more popular. Although in vivo small rodent-based models are still the most common, large animal-based models are used to research region-dependent lung deposition and absorption. Importantly, in vivo rodent, PK data after pulmonary delivery were used to predict human PK and systemic exposures to inhaled drugs utilizing a kinetic modeling technique with allometric scaling. As a result, the J_app_ or P_app_ values of the in vitro pulmonary cell-based models’ fine correlation with in vivo can be used to accurately predict human PK profiles [343]. Additionally, they have yet to be successful in extrapolating to predict the in vivo PK profiles, and this requires more work. In general, the value of preclinical analyses of pulmonary deposition and absorption appears to have shifted more toward their translational capacity to foretell local pulmonary and systemic exposure in humans, as well as to rationalize the best inhaled dosage form and delivery system for the drugs involved. To effectively advance toward product approval and clinical use, it is crucial that scientists and industries make the right selection and timely exploitation of the finest models at each stage of the drug research and development procedure.

## 8. Challenges in Clinical Translation of Liposomes and Extracellular Vesicles

EVs have emerged as an attractive cell-free alternative to state-of-the-art cell therapy. EVs are nanosized particles endogenously produced by cells and serve as a tool for intercellular communication. Currently, EVs are garnering considerable attention, partly due to their role in stem cell paracrine signaling and the ability to epigenetically regulate target cell genes by transporting and releasing their RNA species, such as microRNA [353]. Despite the high potential of EVs to treat variable diseases, the translation into clinical settings needs to overcome several challenges. It is noteworthy that there are not currently approved EVs for clinical use, but there are over 100 EVs products in clinical trials [354]. In comparison, there are currently at least 18 LPs-based drugs approved by the United States Food and Drug Administration (US FDA) or European Medicines Agency (EMA) for clinical use [355]. LPs-related drugs and LPs were approved as early as 1993 (Amphocil^®^ containing Amphotericin B and Epaxal^®^ containing Hepatitis A virus antigen) and 1995 (Doxil^®^ containing Doxorubicin), respectively [355]. This simply denotes the establishment of LPs in clinical translation compared to the EVs and highlights the ability of pharmaceutical manufacturers to overcome the challenges in LPs clinical translation. Despite the success of LPs in drug delivery, there are no LPs currently approved for PDD. The challenges in the clinical translation of EVs and LPs can be viewed from two main points: first, from the pharmacological/PKPD side, and second, from the manufacturing and administration side. From the pharmacological/PKPD side, LPs and EVs have difficulties reaching the target site of action, mainly due to the rapid clearance by MPS and the lack of targeting moiety that can drive the particles to the target cell. When it comes to the PDD, additional challenges are faced due to the difficulty of reaching deep into the alveolar region to give the desired effect. On the other hand, from the manufacturing and administration point of view, the challenges start from the early steps in the preparation into the final steps in transportation and storage. Similar to any emerging delivery system, several challenges come from the unavailability of established methods and instruments for production and characterization. 

### 8.1. Pharmacological/PKPD Challenges

The pharmacological/PKPD challenges are similar to the general challenges of other nanoparticle-based medicines. The outcome of these challenges is mainly seen in the short half-life of EVs and LPs. One study found that plasma–derived small EVs were rapidly cleared from the blood with a half-life of about 7 min [356]. A biodistribution study on EVs was conducted in mice following systemic delivery and revealed that the vesicles accumulated mainly in the liver, spleen, gastrointestinal tract, and lungs. Cell origin, dose, and route of administration affect the biodistribution pattern [324]. Furthermore, different EV subpopulations display different biodistribution patterns [357]. Another study found that cord blood MSC EVs had a plasma half-life of just 1.2–1.3 min [357]. Knowing that the majority of the doses are located in the liver and spleen, a significant amount of the EVs dose is commonly considered lost and not taken up by the target cells. As a result, a higher dose may be required to achieve the therapeutic outcome, which raises safety concerns. An alternative approach is to modify the surface of EVs with a specific ligand such as CD47 to avoid rapid clearance by the immune cells, but more investigations are needed on this approach. The other alternative is to use some established methods in nanomedicine to increase the blood circulation time, similar to stealth LPs. This approach requires modifying the surface with hydrophilic polymers like PEG (PEGylation) or glucose (glycosylation). They can be performed using chemical methods (PEGylation) or molecular methods (glycosylation). Again, these approaches are not yet established for EVs, and their effect on the structure and stability of EVs needs more investigation compared to well-established procedures and knowledge in LP manufacturing. Both EVs and LPs suffer rapid clearance by MPS, and steric stabilization through PEGylation is still the best method to increase blood circulation time. As an example, Doxil^®^ containing doxorubicin, is a PEGylated LPs drug that has already been in the market since 1995 [358].

### 8.2. Manufacturing and Administration Challenges

Under the medicinal products categories from a regulatory perspective, EVs-based products are considered ‘‘biological medicinal products”. Due to the diversity of EVs, EVs-based products might be categorized into different groups from the regulatory perspective. The simplest forms, EVs originating from unmodified primary cells, are considered biological medicinal product categories. Similar consideration might apply to EVs from genetically modified cells with no transgene product. However, it is expected that EVs originating from genetically modified cells with a transgene product to be considered gene therapy products, a subclass of advanced therapy medicinal products (ATMP) [19,359]. Many efforts have been performed to assist in this translation. One of the major contributors is the International Society for Extracellular Vesicles (ISEV). ISEV Task Force on Regulatory Affairs and Clinical Use of EV-based Therapeutics, as well as the Exosomes Committee from the ISCT, are expected to contribute effectively to the development of EV-based medicinal products. This is achieved by providing regulatory bodies with updates on the scientific progress in the EVs field, information to patients, and an expert resource network. A recent paper by ‘Extracellular Vesicle translation to clinical perspectives–EVOLVE France’ demonstrated a good overview of the pathway to develop EV therapeutics with some recommendations at each stage of the development pathway [359]. Taking EVs out of the lab into clinical use requires compliance with all regulatory requirements as stipulated in The International Council for Harmonization of Technical Requirements for Pharmaceuticals for Human Use (ICH) guidelines. For a medicinal product to be registered, a collection of documents needs to be submitted to the regulatory agency. These documents are outlined in ICH Common Technical Document (CTD) regulatory dossier, which was harmonized and adopted by several regulatory agencies all over the world. The requirements are categorized into quality, safety, and efficacy. ICH M4Q explains the quality requirements for registration, which is essential for manufacturers. Following is a more detailed explanation of specific points related to EVs and LPs manufacturing:

#### 8.2.1. Drug Loading and Release

Some challenges come from the early stage of development. Drug loading into EVs needs to be of a pharmaceutical scale to enable the translation into viable medicine. A sufficient quantity of the drug is required to be loaded in order to achieve a therapeutic response. EVs suffer from inherited low loading capacity and low loading efficiency. Loading capacity is referred to the amount of drug that can be loaded in a specific amount of the carrier (EVs), while loading/encapsulation efficiency refers to the percentage of the drug that is loaded into the carrier from the total amount used in the preparation. Because EVs are isolated in their completed bilayer structure, the types of drugs that can be passively loaded are very limited. Meanwhile, biological molecules can be overexpressed in the EV-producing cell leading to a high concentration [358]. Some studies reported inefficient loading and delivery of RNA species [360]. Current reports from published studies revealed variable results. Doxorubicin has loaded into EVs mouse immature dendritic cells at 20% encapsulation efficiency via electroporation [361]. In another study, paclitaxel and doxorubicin were loaded into bEnd. Three cell-derived exosomes at 7.3 ± 1.1 ng and 132.2 ± 2.9 ng per 1 μg, respectively [313]. This is just 0.73% *w*/*w* and 1.3% *w*/*w* loading capacity of paclitaxel and doxorubicin, respectively. In comparison, doxorubicin was loaded in LPs at 90% at the drug-to-lipid ratio of 1:20 *w*/*w,* which is about 4.5% loading capacity [362]. This issue might be more related to the loading of low molecular weight drugs in the EVs other than using engineered EVs. However, the majority of EVs research focuses on using engineering EVs or EVs without a specific payload. It is reported that there are more than 100 exosome-related clinical trials registered at (https://clinicaltrials.gov/ (accessed on 31 December 2022)). Amongst them, the authors reported 13 clinical trials using exosomes as therapeutics, with only one clinical trial using small molecules (curcumin, id: NCT04388982) [354]. In comparison, drug loading into LPs in general easier. Even though the encapsulation efficiency is not straightforwardly high in LPs, there are several methods to increase the encapsulation efficiency, including the remote loading method, the one employed in loading doxorubicin in Doxil^®^ products [363]. Other than loading efficiency, premature drug release is one of the challenges in LPs and, to some extent, EVs. In some cases, LP formulations failed in the clinical trials due to drug release before reaching the site of action [363,364].

#### 8.2.2. Identification and Purity 

For EVs to be translated into clinical use, their based products should comply with the quality requirements. Identification, purity, and potency are among the basic quality attributes for active ingredients that appear more challenging in EVs than small molecules or even protein-based biological products like insulins and monoclonal antibodies. The relationship between EV structure and function is still in the research area [365]. Therefore, defining the critical quality attributes of the final product is yet to be fully established and scientifically justified. One of the main features of EVs, which also contributes to the difficulty in clinical translation, is structure complexity. From chemical aspects, unlike small molecules or even macromolecules, EVs are a complex mixture of chemicals in a specific arrangement. In fact, EVs look more similar to the formulated LPs than active biological ingredients. This means that EVs are active ingredients that look like a formulated drug. In a simplified workflow, EVs-based product preparation starts with cell culture, cell priming (if any), EVs-secretion, EVs harvesting, EVs purification, formulation, filling and finishing, storage, and shipping. From this workflow, it can be considered that the ‘‘Drug Substance” steps start with the cell substrate while the ‘‘Drug Product” steps begin with the formulation [359]. “Drug Substance” refers to the active pharmaceutical ingredient (API), while “Drug Product” refers to the medicinal product in its final form for administration by the patient. Identification is one of the critical aspects of any medicine to be approved for clinical use. For EVs, the exact identity remains unknown or partially known. EVs are known to have proteins, lipids, and nucleic acids, but which one is to be considered the main active ingredient needs to be confirmed. The role of some noncoding RNA species in EVs is not fully understood [353]. It was suggested to perform the identity tests of EVs by hydrodynamic diameter analysis of single particles, immunochemical characterization, and DNA and RNA content [359]. The purity of active pharmaceutical ingredients is another critical aspect to be considered before moving into drug formulation. The ratio between protein content and the number of EVs has been used to express the purity of EVs [359,366]. The purity of EVs is directly related to the isolation technique, which also affects the yield (the number of isolated EVs). Efficacious EVs dose could be quantified using surrogate markers like microRNAs and fingerprint assays to assist in characterizing the purity of EVs [353]. Characterizing the impurity of EVs is more challenging than other active ingredients due to their complex composition and structure. In EVs, purity is not only related to the presence or absence of a specific chemical or ingredient but also to its location of it in the composition. For example, DNA and RNA not encapsulated in EVs may be considered impurities and needs to be analyzed, for example, by quantification using DNase and RNase treatment [359].

#### 8.2.3. Potency

Defining the potency or the biological activity and so on the dose is one important step in translating a research discovery into a clinical medication. The most common way to define the dose of current drugs is the quantity of the active ingredient (e.g., 3 mg/kg/day). For some more complicated definitions, the dose is bio-efficacy based and defined as an international unit (IU), which defines the amount of drug that exerts a specific response. One example of this is the insulin which unit was originally defined as the amount of insulin required to cause convulsive hypoglycemia in a fasted 2 kg rabbit [367]. With respect to EVs, there are several methods to quantify the dose. Commonly reported methods are based on the number of parent cells (cell equivalents), the amount of protein cargo (protein concentration), and the number and size of EVs [368]. Matei et al., 2019, reviewed EVs as a potential therapy for neonatal conditions and then mentioned that the studies they reviewed varied considerably in terms of the dose, frequency, and route of administration of EVs. In addition, the safe and effective dose for neonatal conditions was not determined [353]. In their position paper, Silva et al., 2021, recommended testing the biological activity using a potency test in vitro, if relevant, or else in vivo [359]. It is reported that general guidelines for EVs dosing in vivo are not established yet [369]. The authors performed a meta-analysis of the published studies on in vivo applications of EVs-based therapeutics to determine an effective dose for future efforts, with a total of 64 different pre-clinical studies utilizing EVs as a therapeutic intervention selected. The study revealed a large variation in the dosing of EVs in the pre-clinical studies, where the doses ranged from 0.001 to 100 mg EVs protein per kg body weight, and a similar finding was revealed with the studies that used the particle number as a basis of dosing. The majority of the pre-clinical study relied on the total protein amount as a basis of dosing followed by the total EVs particle number (using resistive pulse sensing (RPS) or nanoparticle tracking analysis (NTA)). On the other hand, the ongoing clinical trials generally used EV particle number as a basis for determining the dose. It is also noteworthy to highlight that applying current dose conversion factors across the species may lead to variable outcomes. A similar observation was seen in PEGylated LPs, where the converted dose from animal models to humans varied by 30% [369,370].

#### 8.2.4. Large-Scale Production

The large-scale production of EVs for use in clinical settings would probably use some established methods currently employed in the biotech industry. Upstream processes like large-scale cell culture using bioreactors and generic downstream processes like centrifugation, TFF, and SEC can be used for EVs isolation. However, new methods may need to be developed for isolating a homogenous population of EVs, for example, affinity-based isolation procedures. The more steps in the manufacturing, the less yield. Therefore, employing several additional steps to obtain a clinically valid and economically viable EV product remains a hurdle. To explain this, a manufacturing process with three steps, each with 90% yield, results in a 72.9% final product. On the other hand, an additional four steps (a total is seven steps) will result in only a 47.8% yield. This accounts for more than 50% loss of an already low amount of EVs that can be finally purified. Production of EVs involves several steps, including cell culture, isolation and purification steps, characterization, and formulation. To ensure process consistency, critical process variables need to be identified and controlled. These variables may include but are not limited to, cell type, number of passages, cell collection process, cell culture conditions, growth media and supplements, type of bioreactor, isolation and storage methods, etc. [365]. These and other variables are known to affect the critical quality attributes of the final product, e.g., EVs composition and size. A meta-analysis study found that the EVs purification method significantly affected the total protein content in the final preparation, which is one of the most critical attributes of the formulation since the EVs dose is frequently calculated based on it. As a result, the study concluded that the doses in the pre-clinical studies that utilized precipitation or ultrafiltration were approximately ten times lower than the studies using size or density purification methods [369]. Another study reported that the number of small EVs was higher when using size-exclusion chromatography as an isolation method compared to ultracentrifugation. The study further concluded that the functional activity of small EVs could depend on the isolation method and may not solely reflect the EV’s quantity [371]. For LPs, large-scale production methods were established for conventional LPs, even without the need for multiple production steps or organic solvents. However, challenges arise when adding functionality to the LPs, like adding targeting ligands [6]. It is not surprising that no clinically approved LP yet are ligand-functionalized [372]. When considering PDD, the manufacturing does not stop with the successful manufacturing of LPs or EVs themselves, but additional steps are needed to formulate them to be suitable for deep deposition in the lungs. The formulation itself is directly related to the inhalation device to be used. As mentioned earlier, DPIs and nebulizers seem to be the most suitable devices for PDD of LPs and EVs.

#### 8.2.5. Process Validation

Consistency and reproducibility of the manufacturing process are core principles in pharmaceutical manufacturing, which are translated in the guidelines as validation and qualification. Good manufacturing practice (GMP) guideline emphasizes that pharmaceutical manufacturing processes need to be validated to ensure that they are able to consistently deliver the same product quality. Conventional dosage forms like tablets and capsules may not have significant process validation problems because the manufacturing processes and equipment are well-defined and established. However, EVs suffer significant challenges in validation whereby the manufacturing processes at a large scale are yet to be established. On the other hand, since there are several LP products already marketed, there should be no significant issues in the validation. However, more validation challenges are expected when employing more complex processes, for example, for ligand-functionalized LPs.

#### 8.2.6. Stability

The stability of EVs is another important concern when moving ‘out from the lab.’ While hundreds of studies focused on EVs in terms of structure, composition, isolation, and others, their studies rarely touch the stability issue. They are usually performed in the lab with freshly-prepared EVs. The stability of EVs needs to be addressed with deeper investigations than conventional dosage forms or even LPs. The size and surface properties are some of the physical aspects that affect EV’s functions. On the other hand, chemical stability requires investigation of the composition of EVs like proteins, lipids, carbohydrates, DNA, and RNA. The effect of storage conditions on these stability aspects is yet to be fully understood. Whether extreme storage conditions like −80 °C are required for the storage and transportation of EVs is still unknown. If such conditions are found to be a necessity for maintaining EVs functions, a spike in the cost will be brought, raising the issue of such therapy being affordable and balancing the benefits. The pharmaceutical industry has already learned from historical lessons when the higher cost is brought with little additional benefits. One of these lessons is the story of the first non-parenteral and innovative insulin product (Exubera^®^). A commercial inhaled insulin product (Exubera^®^) was developed by Sanofi-Aventis and gained approval from the FDA and EMA in 2006 and was marketed by Pfizer. Although it offered the advantage of painless administration by the pulmonary route, it was similar to the subcutaneous (SC) injected rapid-acting insulin in terms of PK/PD characteristics and, thus, offered no additional clinical benefit in postprandial glycemic control. The most important issue, the inhaler device was large with a cumbersome procedure for administration. Therefore, in two years, Pfizer withdrew Exubera^®^ from the market after it failed to gain acceptance from patients and providers (e.g., low sales) [373]. The claimed advantages appeared not to balance the additional cost (+30%) [374]. A similar scenario should be avoided with EVs, i.e., the advantages should be way better than any available treatment to balance the expected higher cost in production, storage, and administration. 

Several studies reported variable stability aspects of the EVs, but in general, the stability studies were conducted for short durations (hours to days), and many found a reduction in EV’s protein content or activity [375]. On the other hand, other studies found that the storage of plasma at 4, −20, or −80 °C did not result in significant degradation of EVs-associated RNA. The stability of the EVs appeared to be related to the sample origin, with −80 °C as the most promising storage condition [375]. Such a storage temperature adds more complications to the clinical translation of EVs. Therefore, it is not surprising to know that there was no agreement among the scientists about the storage and stability of EVs during a survey conducted by the International Society for Extracellular Vesicles (ISEV) in 2018 [376]. On the other hand, LPs formulations are prone to several stability issues, such as phospholipid hydrolysis and peroxidation and the ability to reconstitute after lyophilization. These issues are also related to EVs since they have a relatively similar general structure to LPs. However, they are less complicated and challenging in LPs since the lipid composition, and the other components of the formulation are all known and well-studied. In contrast, EVs composition is more complicated in terms of lipids and other ingredients, and the manufacturer does not have full control over that, unlike in LPs.

## 9. Opinion: Suitability of Liposomes and Extracellular Vesicles for Pulmonary Drug Delivery, Comparison and Proposed Solutions to Overcome the Challenges

Drug formulations that are designed for PDD require specific characteristics with special attention to the particle size. The particle size to consider here is the mass median particle size, which might differ significantly from the particle size measured using the common methodologies used in nanoparticle searches like photon correlation spectroscopy, NTA, and microscopy techniques. The suitable size for deep deposition is around 1–3 µm, far above the normal particle size of EVs and LPs. Therefore, both EVs and LPs need to be delivered at a larger size, which can be achieved either by loading them onto a solid carrier (e.g., during lyophilization) or through small droplets. Unlike most drug delivery routes, PDD is achieved using devices. Therefore, it is always important to consider both formulation and device. DPIs and nebulizers seem to be the best options for delivering LPs and EVs. The DPIs require solid-state formulation, while the nebulizers accept both solid and liquid formulations, but in the end, the drug is nebulized in a liquid form.

In order to make a solid formulation of LPs and EVs, lyophilization is usually considered. Lyophilization is one option to increase the stability of nanoparticles. However, it is more challenging for both EVs and LPs because they have fragile membrane structures. In many cases, LPs are formulated as pro-LPs in the dry powder dosage form then they will be converted into LPs once the powder comes in contact with the lungs’ fluids. EVs seem to lack this possibility since they are manufactured by the cells in their ready bilayer vesicle form. On the other hand, nebulization is another potential method to deliver EVs and LPs through PDD. In this method, there is no need to subject the particles to a dehydration step like in the lyophilization, even though it is also possible to lyophilize the particles and then resuspend them before use. Therefore, the particles might have better stability in terms of physical characteristics like particle size and coagulation. However, the particles need to be stored in their suspension (liquid) form. Being in a solution, the particles may be subjected to immature release of the active ingredients before administration. The premature release might be of great concern for drugs loaded into LPs and EVs but of less damage for the BioEVs, whereby the active ingredients are already part of the EVs structure.

From a manufacturing point of view, both LPs and EVs need to overcome the GMP manufacturing requirements. However, LPs are more established in large-scale manufacturing and are already marketed. Therefore, LPs are less challenging to be manufactured on a large scale. For PDD, additional manufacturing steps are needed to make the formulation suitable for PDD, especially for the particle size requirements. These steps are established for conventional drugs but not yet for LPs or EVs. LP preparation as pro-LPs that can be delivered using DPI looks like a viable option for large-scale manufacturing and long-term stability. In addition, it can also be used in nebulizers. LPs also can be manufactured in liquid dosage form and then added to the nebulizers directly before administration. However, for EVs, the formulation as liquid dosage form and then using the nebulizers as the delivery device seems to be the best option. When considering the type of drug to be delivered, LPs seem to be more practical for small molecules, whereas EVs are suggested to be reserved for the potent, complex drugs through bioengineering of the producing cells. In this regard, hybrid vesicles that can be produced by fusing both LPs and EVs emerge as a new platform to deliver a wider range of drugs. For the drugs that can be easily encapsulated in LPs, fusing with EVs add some features like the surface properties of EVs. On the other hand, for the bioengineered drugs in EVs, fusion with LPs help to overcome some of the EV limitations and make the formulation more controlled in term of composition. 

## 10. Conclusions

LPs and EVs are lipid nanoparticles with several similarities in terms of structure, lipid composition, ability to load and protect drugs, particle size, PKPD, and others. On the other hand, they are distinguished in their origin, method of manufacturing, and composition complexity. The lack of established large-scale manufacturing equipment and processes makes EVs more difficult to be commercialized than LPs. When considering PDD, several challenges are added to translating LPs and EVs to clinical use. Since PDD is associated with delivery devices, DPIs can be used for LP delivery, mostly as pro-LPs, whereas nebulizers can suit both LPs and EVs. In order to take the best of both and reduce the limitations; hybrid vesicles are proposed as a new delivery vehicle that can be explored for PDD.

## Figures and Tables

**Table 3:** Summary of in vitro pulmonary cell-based models for testing inhaled aerosolized mist/dried lipid bilayer vesicles.

In Vitro Model	Model Characteristic	Human Physiological Realism-In Vivo Correlation
LLI: liquid-liquid interface	Submerged cultured cells exposure to vesicles dissolution or suspension in the culture medium	Unrealistic artificial alveolar environment incapable of real-time cell-delivered vesicles dosimetry
ALI: air-liquid interface	Vesicles deposition onto the cultured cells exposed to the inhaled air	Realistic physiologically alveolar relevant environment capable of real-time cell-delivered vesicles dosimetry
ALICE-CLOUD: air-liquid interface cell exposure-cloud (e.g., VITROCELL^®^) empowered with quartz crystal microbalances (QCMs) [337]	Aerosols cloud generates by nebulization gently deposits onto the cultured cells due to single particle sedimentation and cloud setting	Highly realistic physiologically alveolar relevant environment capable of real-time cell-delivered vesicles dosimetry

**Table 4:** Summary of ex vivo lung tissue/organ-based models for testing inhaled aerosolized mist/dried lipid bilayer vesicles.

Ex Vivo Model	Model Characteristic	Human Physiological Realism-In Vivo Correlation
IPRL: isolated perfused rat lung	Preserves lung architecture and function without confounding whole-body complications	Allow the in vivo-relevant lung tissue/organ-level kinetic assessment of absorption and deposition
IPRL-QSAR: isolated perfused rat lung-quantitative structure activity relationship (In Silico, i.e., Computer Simulation)	Improve compounds design via predicting pulmonary absorption	Replace the routine generation of IPRL model data for ranking and classifying compounds prior to synthesis
IPRL-PBBP: isolated perfused rat lung-physiologically based biopharmaceutical dissolution data (In Silico, i.e., Computer Simulation)	Improve pulmonary drug absorption understanding via comparing absorption rates of poorly soluble inhaled drugs from suspensions and dry powders with solutions	A useful tool for investigating and improving pulmonary dissolution of poorly soluble inhaled drugs
IPRL-IV PK: isolated perfused rat lung-intravenous pharmacokinetics (In Silico, i.e., Computer Simulation)	Improves extent predictability using input parameters from IPRL ex vivo model	IPRL ex vivo (not in vitro) data are better to understand how different drug properties and formulation might affect in vivo behavior of inhaled compounds

**Table 5:** Summary of in vivo whole animal-based models for testing inhaled aerosolized mist/dried lipid bilayer vesicles.

In Vivo Model	Model Characteristic	Human Physiological Realism-In Vivo Correlation
Non-surgical orotracheal administration	Spray-instilled as solution or suspension or insufflated as powders	Predict inhaled drugs human PK profiles from in vivo rodent PK data
Intratracheal powder insufflation	Spray-insufflated as powders	Predicted human PK profiles by kinetic modeling requires further systematic refinement
Intratracheal solution delivery	Spray- instilled as solution	Predicted human PK profiles by translational compartmental modeling are remarkably identical

## Data Availability

Not applicable.
